# Bypassing the lattice BCS–BEC crossover in strongly correlated superconductors through multiorbital physics

**DOI:** 10.1038/s41535-024-00706-7

**Published:** 2024-12-10

**Authors:** Niklas Witt, Yusuke Nomura, Sergey Brener, Ryotaro Arita, Alexander I. Lichtenstein, Tim O. Wehling

**Affiliations:** 1 I. Institute of Theoretical Physics, University of Hamburg, Notkestraße 9-11, 22607 Hamburg, Germany; 2 The Hamburg Centre for Ultrafast Imaging, Luruper Chaussee 149, 22607 Hamburg, Germany; 3 Institute for Materials Research (IMR), Tohoku University, 2-1-1 Katahira, Aoba-ku, Sendai, 980-8577 Japan; 4 Department of Physics, The University of Tokyo, 7-3-1 Hongo, Bunkyo-ku, Tokyo 113-0033 Japan; 5 RIKEN Center for Emergent Matter Science (CEMS), 2-1 Hirosawa, Wako, Saitama 351-0198 Japan

**Keywords:** Superconducting properties and materials, Bose-Einstein condensates, Theory and computation

## Abstract

Superconductivity emerges from the spatial coherence of a macroscopic condensate of Cooper pairs. Increasingly strong binding and localization of electrons into these pairs compromises the condensate’s phase stiffness, thereby limiting critical temperatures – a phenomenon known as the BCS–BEC crossover in lattice systems. In this study, we demonstrate enhanced superconductivity in a multiorbital model of alkali-doped fullerides (A_3_C_60_) that goes beyond the limits of the lattice BCS–BEC crossover. We identify that the interplay of strong correlations and multiorbital effects results in a localized superconducting state characterized by a short coherence length but robust stiffness and a domeless rise in critical temperature with increasing pairing interaction. To derive these insights, we introduce a new theoretical framework allowing us to calculate the fundamental length scales of superconductors, namely the coherence length (*ξ*_0_) and the London penetration depth (*λ*_L_), even in presence of strong electron correlations.

## Introduction

The collective and phase coherent condensation of electrons into bound Cooper pairs leads to the emergence of superconductivity. This macroscopic coherence enables dissipationless charge currents, perfect diamagnetism, fluxoid quantization, and technical applications^1,2^ ranging from electromagnets in particle accelerators to quantum computing hardware. Often, superconducting (SC) functionality is controlled by the critical surface spanned by critical magnetic fields, currents, and temperatures which a SC condensate can tolerate. Fundamentally, these are determined by the characteristic length scales of a superconductor – the London penetration depth, *λ*_L_, and the coherence length, *ξ*_0_.

*λ*_L_ and *ξ*_0_ quantify different aspects of the SC condensate: The penetration depth is the length associated with the mass term that the vector potential gains through the Anderson-Higgs mechanism^3^. In consequence, magnetic fields decay exponentially over a distance *λ*_L_ inside a superconductor. Through this, *λ*_L_ is connected to the energy cost of order parameter (OP) phase variations and hence the SC stiffness *D*_s_. The coherence length, on the other hand, is the intrinsic length scale of OP amplitude variations and is associated with the amplitude Higgs mode. *ξ*_0_ sets the scale below which amplitude and phase modes significantly couple such that spatial variations of the OP’s phase reduce its amplitude^3,4^.

**Fig. 1:**
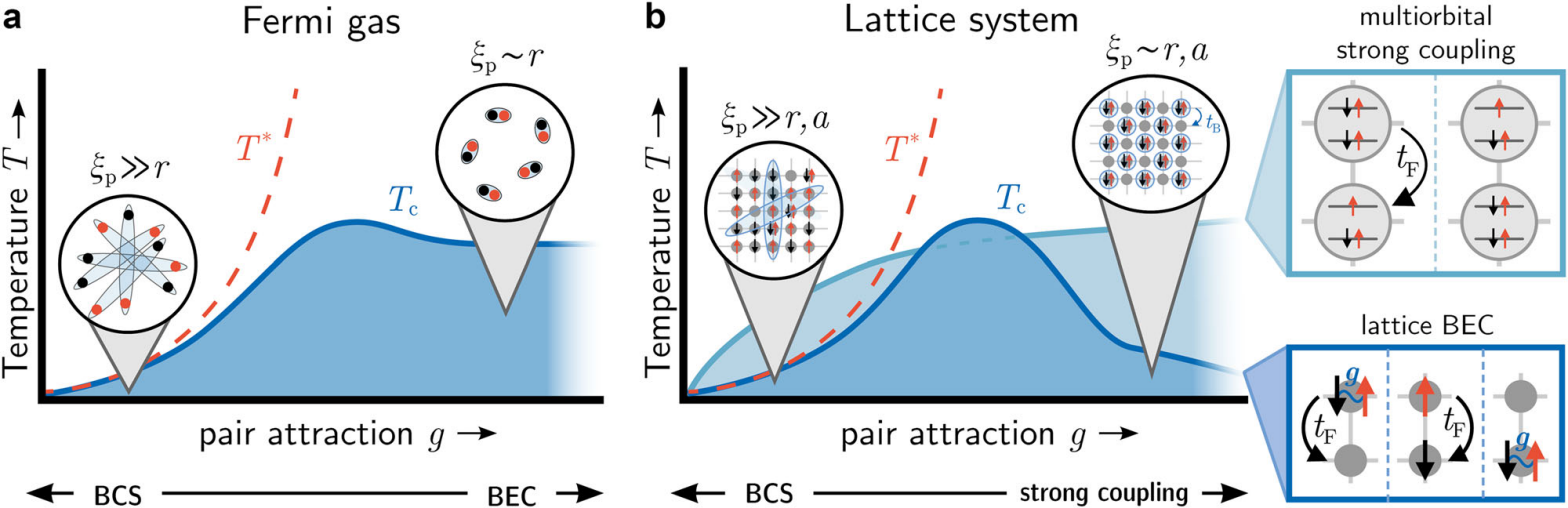
BCS–BEC crossover in Fermi gases vs. lattice systems. Evolution of the critical temperature *T*_c_ and Cooper pair size *ξ*_p_ in the BCS–BEC crossover (dark blue line) for (**a**) Fermi gases and (**b**) lattice systems. Both cases display a dome-shaped behavior of *T*_c_ in the intermediate crossover regime but behave qualitatively different in the strong coupling BEC phase: *T*_c_ remains finite in the Fermi gas (**a**)^31^ but approaches zero in the lattice case (**b**). During the crossover, *ξ*_p_ is reduced to the order of interparticle spacing *r* (**a**, **b**) and lattice constant *a* (**b**). For the lattice system, we contrast the evolution towards the multiorbital strong coupling phase (light blue line) discussed in this article. Here, the localization of pairs differently affects the bosonic hopping *t*_B_, as drawn in the insets on the right. In the lattice BEC limit, bosonic hopping relies on a second-order process involving two fermionic hoppings, *t*_F_, and a virtual intermediate state with broken pairs that is inhibited by the strong attraction *g* < 0. Consequently, this process and also ${T}_{{\rm{c}}}\propto {t}_{{\rm{B}}}={t}_{{\rm{F}}}^{2}/| g|$ are quenched at large ∣*g*∣^21,24^. In the multiorbital strong coupling case, the local coexistence of paired and unpaired electrons fluctuating between different orbitals enables bosonic hopping *t*_B_ as a first order process in *t*_F_ without any intermediate broken pair states. See Supplementary Note 7 for details on the involved states and energy diagram specific to the case of A_3_C_60_. A second temperature scale T* is drawn in both panels as its splitting from *T*_c_ (corresponding to the opening of a pseudogap) marks the beginning of the crossover regime^24^.

In addition to influencing the macroscopic properties of superconductors, *λ*_L_ and *ξ*_0_ play an important role to understand strongly correlated superconductors, as is epitomized in the Uemura plot^5–8^. For instance, the interplay of *λ*_L_ and *ξ*_0_ impacts critical temperatures^9^, it is relevant for the pseudogap formation^10–13^, it influences magneto-thermal transport properties like the Nernst effect^8,14,15^, and it might underlie the light-enhancement of superconductivity^15–19^. An important concept in this context is the BCS–BEC crossover phenomenology^20–24^. It continuously connects the two limiting cases of weak-coupling Bardeen–Cooper–Schrieffer (BCS) superconductivity with weakly-bound and largely overlapping Cooper pairs to tightly-bound molecule-like pairs in the strong-coupling Bose–Einstein condensate (BEC) as the interaction strength or the density is varied (Fig. 1).

The BCS–BEC crossover has been studied in ultracold Fermi gases^23^, low-density doped semiconductors^25^, and is under debate for several unconventional superconductors^6,8,24,26–30^. However, quasi-continuous systems, for instance Fermi gases, and strongly correlated superconducting solids show a crucially different behavior of how their SC properties, most importantly the critical temperature *T*_c_, change towards the strong coupling BEC limit. While *T*_c_ converges to a constant temperature *T*_BEC_ for Fermi gases in a continuum^31^ (Fig. 1a), *T*_c_ can become arbitrarily small in strongly correlated lattice systems due to the quenching of kinetic energy (Fig. 1b). Since the movement of electron pairs, i.e., bosonic hopping, necessitates intermediate fermionic hopping, it becomes increasingly unfavorable for strong attractions. Thus, as Cooper pairs become localized on the scale of the lattice constant, the condensate’s stiffness and hence *T*_c_ are compromised^21,24^. Figure 1 contrasts this generic BCS–BEC crossover picture for Fermi gases and correlated lattice systems in terms of the change of *T*_c_ and the pair size *ξ*_p_ as a function of pairing strength. Due to the decrease of *T*_c_ in the BCS and BEC limits, a prominent dome-shape of *T*_c_ can be expected in the crossover region for solid materials. Because of this, recent experimental efforts to increase *T*_c_ concentrate on stabilizing SC materials in this region^24,25,27,30^.

In this work, we demonstrate how multiorbital effects^32,33^ can enhance superconductivity beyond the expectations of the lattice BCS–BEC crossover phenomenology (as contrasted in Fig. 1b) with a model inspired by alkali-doped fullerides (A_3_C_60_ with A = K, Rb, Cs). The material family of A_3_C_60_ hosts exotic *s*-wave superconductivity of critical temperatures up to *T*_c_ = 38 K, being the highest temperatures among molecular superconductors^34–37^, and they possibly reach photo-induced SC at even higher temperatures^15,17,18,38^. In order to theoretically characterize the SC state, the knowledge of the intrinsic SC length scales is essential. While BCS theory and Eliashberg theory provide a microscopic description of *λ*_L_ and *ξ*_0_ for weakly correlated materials^39–41^, their validity is unclear for superconductors with strong electron correlations. To the best of our knowledge, *ξ*_0_ is generally not known from theory in strongly correlated materials. Only approaches to determine *λ*_L_ exist where an approximate, microscopic assessment of the SC stiffness from locally exact theories has been established, albeit neglecting vertex corrections^42–47^.

For this reason, we introduce a novel theoretical framework to microscopically access *λ*_L_ and *ξ*_0_ from the tolerance of SC pairing to spatial OP variations. Central to our approach are calculations in the superconducting state under a constraint of finite-momentum pairing (FMP). Via Nambu–Gor’kov Green functions we get direct access to the superconducting OP and the depairing current *j*_dp_ which in turn yield *ξ*_0_ and *λ*_L_. The FMP constraint is the SC analog to planar spin spirals applied to magnetic systems^48–50^. As in magnetism, a generalized Bloch theorem holds that allows us to consider FMP without supercells; see Supplementary Note 2 for a proof. As a result, our approach can be easily embedded in microscopic theories and ab initio approaches to tackle material-realistic calculations.

In this work, we implement FMP in Dynamical Mean-Field theory (DMFT)^51^ to treat strongly correlated superconductivity. In DMFT, the interacting many-body problem is solved by self-consistently mapping the lattice model onto a local impurity problem. By this, local correlations are treated exactly. We apply the FMP-constrained DMFT to A_3_C_60_ where we find *ξ*_0_ and *λ*_L_ in line with experiment for model parameter ranges derived from ab initio estimates, validating our approach. For enhanced pairing interaction, we then reveal a multiorbital strong coupling SC state with minimal *ξ*_0_ on the order of only 2 – 3 lattice constants, but with robust stiffness *D*_s_ and high *T*_c_ which increases with the pair interaction strength without a dome shape. This strong coupling SC state is distinct to the lattice BCS–BEC crossover phenomenology showing promising routes to optimize superconductors with higher critical temperatures. We discuss this possibility in-depth after the presentation of our results.

The remaining paper is organized as follows: First, we motivate how the FMP constraint is linked to *λ*_L_ and *ξ*_0_ from phenomenological Ginzburg–Landau theory after which we summarize the microscopic approach from Green function methods. Technical details are available in the Supplementary Information as cross-referenced at relevant points. Subsequently, we discuss our results for the multiorbital model of A_3_C_60_. Readers primarily interested in the analysis of these results might skip the first part on the physical motivation for obtaining superconducting length scales from the FMP constraint.

## Results

### Extracting superconducting length scales from the constraint of finite-momentum pairing

**Fig. 2:**
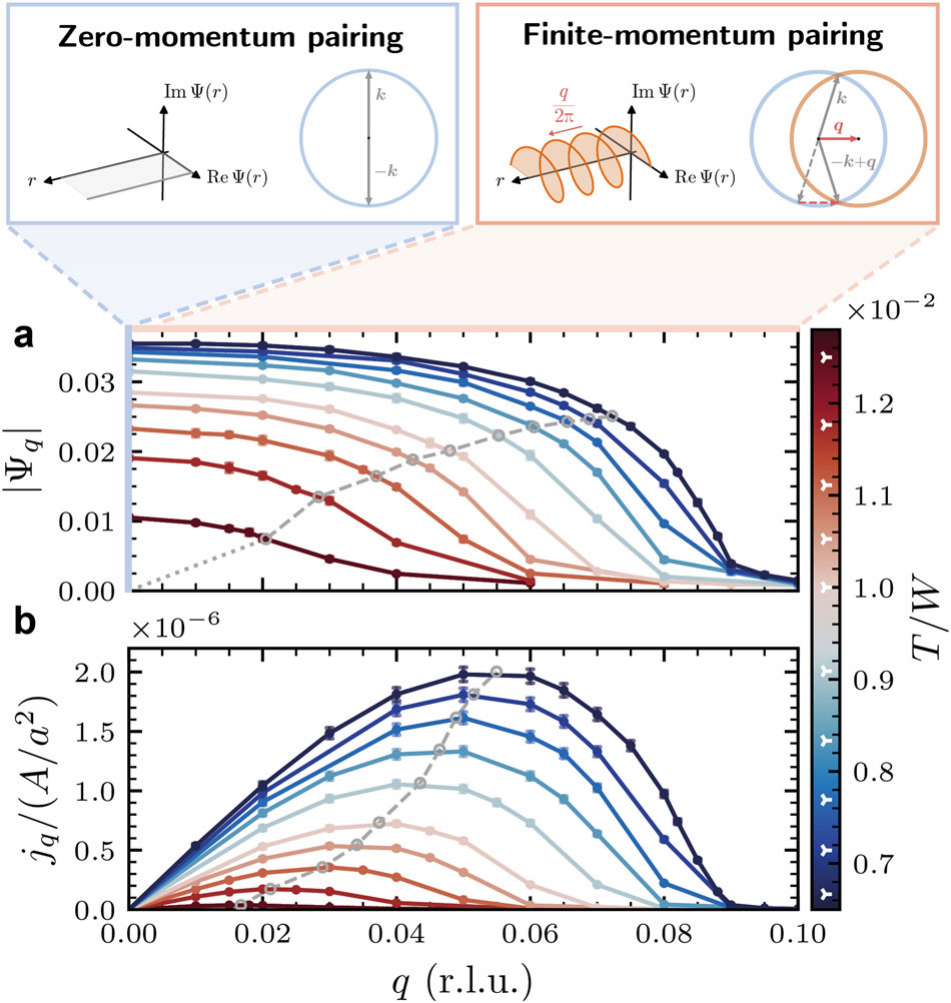
Influence of finite-momentum pairing (FMP) constraint on the superconducting condensate. The top panel insets sketch the position and momentum space representation of the order parameter (OP) Ψ_***q***_(***r***) = ∣Ψ_***q***_∣e^*i****qr***^ in the zero-momentum (left, *q* = 0) and finite-momentum pairing states (right, *q* > 0). The main panels show (**a**) the momentum dependence of the OP modulus and (**b**) the supercurrent density *j*_*q*_ = ∣***j***_***q***_∣ as function of Cooper pair momentum *q* = ∣***q***∣ in reciprocal lattice units (r.l.u.). Gray lines indicate the points of extracting *ξ* and *j*_dp_ (see text). The data shown are results for the A_3_C_60_ model (c.f. Eq. (7)) with lattice constant *a* and interaction parameters *U*/*W* = 1.4, *J*/*W* = − 0.04 evaluated at different temperatures *T* (color coded; see color bar with white triangular markers).

In most SC materials, Cooper pairs do not carry a finite center-of-mass momentum ***q*** = **0**. Yet, in presence of external fields, coexisting magnetism, or even spontaneously SC states with FMP, i.e., ***q*** ≠ **0**, might arise^52–56^ as originally conjectured in Fulde–Ferrel–Larkin–Ovchinnikov (FFLO) theory^57–59^. Here, we introduce a method to access the characteristic SC length scales *ξ*_0_ and *λ*_L_ of strongly correlated materials through the calculation of FMP states with a manually constrained pair center-of-mass momentum ***q***^4,40,60–62^. For this, we enforce the OP to be of the FMP form Ψ_***q***_(***r***) = ∣Ψ_***q***_∣e^*i****qr***^ corresponding to FF-type pairing^57^, which is to be differentiated from pair density waves with amplitude modulations^58,59,63^. We contrast the OP for zero and finite momentum in real and momentum space in the top panel of Fig. 2. For FMP, the OP’s phase is a helix winding around the direction of ***q***, while the OP for zero-momentum pairing is simply a constant.

Before turning to the implementation in microscopic approaches, we motivate how the FMP constraint relates to *λ*_L_ and *ξ*_0_. The Ginzburg–Landau (GL) framework provides an intuitive picture to this connection^4,40,61^ which we summarize here and discuss in detail in Supplementary Note 1, including a derivation of the following equations. The GL low-order expansion of the free energy density *f*_GL_ in terms of the FMP-constrained OP reads

1 $${f}_{{\rm{GL}}}[{\Psi }_{{\boldsymbol{q}}}]=\alpha | {\Psi }_{{\boldsymbol{q}}}{| }^{2}+\frac{b}{2}| {\Psi }_{{\boldsymbol{q}}}{| }^{4}+\frac{{\hslash }^{2}}{2{m}^{* }}{q}^{2}| {\Psi }_{{\boldsymbol{q}}}{| }^{2}$$

with *q* = ∣***q***∣. *α*, *b*, and m* are the material and temperature dependent GL parameters. Here, the temperature-dependent (GL) correlation length *ξ*(*T*) appears as the natural length scale of the amplitude mode (∝∣*α*∣) and the kinetic energy term

2 $$\xi (T)=\sqrt{\frac{{\hslash }^{2}}{2{m}^{* }| \alpha | }}={\xi }_{0}{\left(1-\frac{T}{{T}_{{\rm{c}}}}\right)}^{-\frac{1}{2}}$$

with its zero-temperature value *ξ*_0_ being the coherence length^4^. The stationary point of Eq. (1) shows that the ***q***-dependent OP amplitude $\left\vert {\Psi }_{{\boldsymbol{q}}}\right\vert =\left\vert {\Psi }_{0}\right\vert \sqrt{1-{\xi }^{2}{q}^{2}}$ (*T*-dependence suppressed) decreases with increasing momentum *q*. For large enough *q*, SC order breaks down (i.e., Ψ_***q***_ → 0) as the kinetic energy from phase modulations becomes comparable to the gain in energy from pairing. The length scale associated with this breakdown is *ξ*(*T*) and can, therefore, be inferred from the *q*-dependent OP suppression. We employ, here, the criterion $\xi (T)=1/(\sqrt{2}| {\boldsymbol{Q}}|)$ with ***Q*** such that $| {\Psi }_{{\boldsymbol{Q}}}(T)/{\Psi }_{0}(T)| =1/\sqrt{2}$ for fixed *T* (see Supplementary Note 5-A for more information).

The finite center-of-mass momentum of the Cooper pairs entails a charge supercurrent ***j***_***q***_ ∝ ∣Ψ_***q***_∣^2^***q***, c.f. Eq. (8) in Supplementary Note 1. This current density is a non-monotonous function of *q* with a maximum called depairing current density *j*_dp_. It provides a theoretical upper bound to critical current densities, *j*_c_, measured in experiment. We note that careful design of SC samples is necessary for *j*_c_ reaching *j*_dp_ as its value crucially depends on sample geometry and defect densities^40,61,64^. As the current is related to the London penetration depth *λ*_L_ through the second London equation ${\mu }_{0}{\boldsymbol{j}}=-{\lambda }_{{\rm{L}}}^{-2}{\boldsymbol{A}}$, we can determine *λ*_L_ from *j*_dp_ in GL theory via

3 $${\lambda }_{{\rm{L}}}(T)=\sqrt{\frac{{\Phi }_{0}}{3\sqrt{3}\pi {\mu }_{0}\xi (T){j}_{{\rm{dp}}}(T)}}={\lambda }_{{\rm{L}},0}{\left(1-{\left(\frac{T}{{T}_{{\rm{c}}}}\right)}^{4}\right)}^{-\frac{1}{2}}$$

with the magnetic flux quantum *Φ*_0_ = *h*/2*e*. The temperature dependence with the quartic power stated here is empirical^39,40^ and we find that it describes our calculations better than the linearized GL expectation as discussed in Supplementary Note 5-B. Note that taking the *q* → 0 limit in our approach is related to linear-response-based methods to calculate *λ*_L_ or, equivalently, the stiffness *D*_s_^47,62^.

The GL analysis shows that the OP suppression and supercurrent induced by the FMP constraint connect to *ξ*_0_ and *λ*_L_. In a microscopic description, we acquire the OP and supercurrent density from the Nambu–Gor’kov Green function

4 $$\begin{array}{lll} {\mathcal{G}}_{{\boldsymbol{q}}}(\tau,{\boldsymbol{k}}) = -\langle T_{\tau}\psi_{{\boldsymbol{k}},{\boldsymbol{q}}}(\tau)\psi^{\dagger}_{{\boldsymbol{k}},{\boldsymbol{q}}}\rangle \\\qquad\qquad\quad =\left( \begin{array}{ll} G_{{\boldsymbol{q}}}(\tau,{\boldsymbol{k}}) &F_{{\boldsymbol{q}}}(\tau,{\boldsymbol{k}})\\ F^{*}_{{\boldsymbol{q}}}(\tau,{\boldsymbol{k}}) &{\bar{G}}_{{\boldsymbol{q}}}(\tau,-{\boldsymbol{k}}) \end{array}\right) \end{array}$$

where ${\psi }_{{\boldsymbol{k}},{\boldsymbol{q}}}^{\dagger }=({c}_{{\boldsymbol{k}}+\frac{{\boldsymbol{q}}}{2}\uparrow }^{\dagger }\,\,{c}_{-{\boldsymbol{k}}+\frac{{\boldsymbol{q}}}{2}\downarrow })$ (orbital indices suppressed) are Nambu spinors that carry an additional dependence on ***q*** due to the FMP constraint. *G* (*F*) denotes the normal (anomalous) Green function component for electrons (*G*) and holes ($\bar{G}$) in imaginary time *τ*. For *s*-wave superconductivity as in A_3_C_60_^65–68^, we use the orbital-diagonal, local anomalous Green function as the OP

5 $$| {\Psi }_{{\boldsymbol{q}}}| \equiv {[{F}_{{\boldsymbol{q}}}^{{\rm{loc}}}(\tau = {0}^{-})]}_{\alpha \alpha }=\sum _{{\boldsymbol{k}}}\langle {c}_{\alpha {\boldsymbol{k}}+\frac{{\boldsymbol{q}}}{2}\uparrow }{c}_{\alpha -{\boldsymbol{k}}+\frac{{\boldsymbol{q}}}{2}\downarrow }\rangle \ ,$$

which is the same for all orbitals *α*. This allows us to work with a single-component OP. The current density can be calculated via (c.f. Eq. (26) in the Supplementary Information)

6 $${{\boldsymbol{j}}}_{{\boldsymbol{q}}}=\frac{2e}{{N}_{{\boldsymbol{k}}}}\sum _{{\boldsymbol{k}}}{{\rm{Tr}}}_{\alpha }\left[\underline{{\boldsymbol{v}}}({\boldsymbol{k}}){\underline{G}}_{{\boldsymbol{q}}}\left(\tau ={0}^{-},{\boldsymbol{k}}-\frac{{\boldsymbol{q}}}{2}\right)\right]$$

where $\hslash \underline{{\boldsymbol{v}}}={\nabla }_{{\boldsymbol{k}}}\underline{h}({\boldsymbol{k}})$ is the group velocity obtained from the one-electron Hamiltonian $\underline{h}({\boldsymbol{k}})$ and the trace runs over the orbital indices of $\underline{{\boldsymbol{v}}}$ and ${\underline{G}}_{{\boldsymbol{q}}}$. Underlined quantities indicate matrices in orbital space, *N*_***k***_ is the number of momentum points, and *e* is the elementary charge. See Methods and Supplementary Note 4 for details on the DMFT-based implementation and Supplementary Notes 3 for a derivation and discussion of Eq. (6).

The bottom panels of Fig. 2 show an example of our DMFT calculations which illustrates the ***q***-dependence of the OP amplitude and current density for different temperatures *T*. Throughout the paper, we choose ***q*** parallel to a reciprocal lattice vector ***q*** = *q****b***_1_. We find a monotonous suppression of the OP with increasing *q*. The supercurrent initially grows linearly with *q*, reaches its maximum *j*_dp_ and then collapses upon further increase of *q*. Thus, both ∣Ψ_*q*_∣ and *j*_*q*_ behave qualitatively as expected from the GL description. For decreasing temperature, the point where the OP gets significantly suppressed moves towards larger momenta *q* (smaller length scales *ξ*(*T*)), while *j*_dp_(*T*) increases. We indicate the points where we extract *ξ*(*T*) and *j*_dp_(*T*) with gray circles connected by dashed lines.

### Superconducting coherence in alkali-doped fullerides

We apply FMP superconductivity to study a degenerate three-orbital model *H* = *H*_kin_ + *H*_int_, where *H*_kin_ is the kinetic energy and the electron-electron interaction is described by a local Kanamori–Hubbard interaction^69^

7 $${H}_{{\rm{int}}}=(U-3J)\frac{\hat{N}(\hat{N}-1)}{2}-J\left(2{\hat{{\boldsymbol{S}}}}^{2}+\frac{1}{2}{\hat{{\boldsymbol{L}}}}^{2}-\frac{5}{2}\hat{N}\right)$$

with total number $\hat{N}$, spin $\hat{{\boldsymbol{S}}}$, and angular momentum operator $\hat{{\boldsymbol{L}}}$. The independent interactions are the intraorbital Hubbard term *U* and Hund exchange *J*, as we use the SU(2) × SO(3) symmetric parametrization. This model is often discussed in the context of Hund’s metal physics relevant to, e.g., transition-metal oxides like ruthenates with partially filled *t*_2*g*_ shells^69,70^. In the special case of negative exchange energy *J* < 0, it has been introduced to explain superconductivity in A_3_C_60_ materials^65–68^. In fullerides, exotic *s*-wave superconductivity exists in proximity to a Mott-insulating (MI)^35,36,71^ and a Jahn–Teller metallic phase^36,72–74^. The influence of strong correlation effects and inverted Hund’s coupling were shown to be essential for the SC pairing^65–68,75^ utilizing orbital fluctuations^76^ in a Suhl–Kondo mechanism^72^. In the weakly correlated regime of *U* → 0 and small *J*, the system follows BCS phenomenology^66^.

The inversion of *J* seems unusual from the standpoint of atomic physics, where it dictates the filling of atomic shells via Hund’s rules. In A_3_C_60_, a negative *J* is induced by the electronic system coupling to intramolecular Jahn–Teller phonon modes^68,75,77,78^. As a result, Hund’s rules are inverted such that states which minimize first spin ***S*** and then angular momentum ***L*** are energetically most favorable, see the second term of Eq. (7) for *J* < 0.

**Fig. 3:**
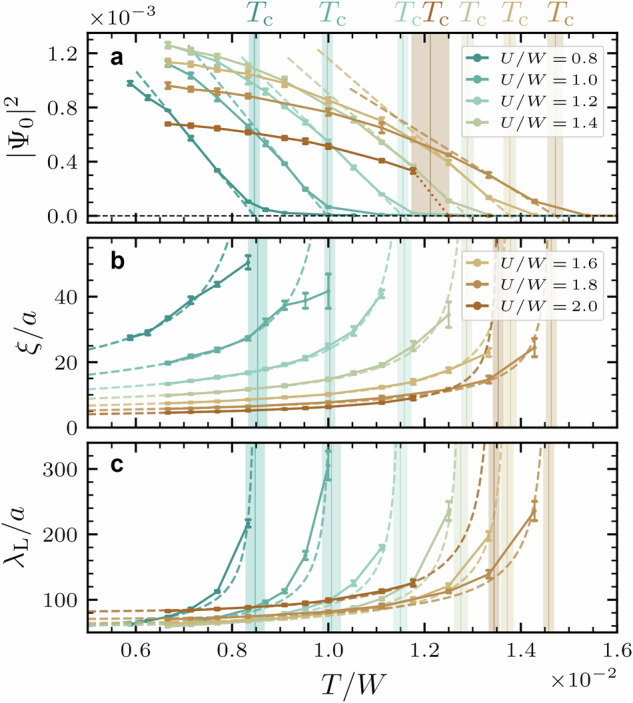
Order parameter, correlation length, and penetration depth in A_3_C_60_. The temperature dependence of (**a**) the zero-momentum order parameter ∣Ψ_0_∣, (**b**) the correlation length *ξ*, and (**c**) the London penetration depth *λ*_L_ are shown for different ratios *U*/*W*. The data were obtained for fixed *J*/*W* = − 0.04 as estimated from ab initio data. Length scales are given in units of the lattice constant *a*. Fits to extract critical temperatures *T*_c_ and zero-temperature values *ξ*_0_ and *λ*_L,0_ are shown by dashed lines. The region of uncertainty to fit *T*_c_ is indicated by shaded regions.

We connect the model to A_3_C_60_ by using an ab initio derived model for the kinetic energy^79^

8 $${H}_{{\rm{kin}}}=\sum\limits_{ij}\sum\limits_{\alpha \gamma \sigma }{t}_{\alpha \gamma }({{\boldsymbol{R}}}_{ij}){c}_{i\alpha \sigma }^{\dagger }{c}_{j\gamma \sigma}$$

where *t*_*αγ*_(***R***_*ij*_) is the hopping amplitude between half-filled *t*_1*u*_ orbitals (*α*, *γ* = 1, 2, 3) of C_60_ molecules on sites connected by lattice vector ***R***_*ij*_. We take the bandwidth *W* as the unit of energy (*W* ≈ 0.3 – 0.5 eV for Cs to K based A_3_C_60_)^68,79^, see Supplemental Note 4-D for further details. For the interaction, we take the first principles’ estimates of fixed *J*/*W* = − 0.04 and varying *U*/*W*^65–68,73^ to emulate unit cell volumes as resulting from the size of different alkali dopants. We solve this Hamiltonian using DMFT, which explicitly takes into account superconducting order. Through the momentum dependence of ∣Ψ_***q***_(*T*)∣ and ***j***_***q***_(*T*), *ξ*(*T*) and *λ*_L_(*T*) can be extracted as discussed in the previous subsection. We show the derived temperature dependence of ∣Ψ_*q*=0_(*T*)∣, *ξ*(*T*), and *λ*_L_(*T*) for different *U*/*W* in Fig. 3.

Close to the transition point, the OP vanishes and the critical temperature *T*_c_ can be extracted from ∣Ψ_0_(*T*)∣^2^ ∝ *T* − *T*_c_. We find that *T*_c_ increases with *U*, contrary to the expectation that a repulsive interaction should be detrimental to electron pairing. This behavior is well understood in the picture of strongly correlated superconductivity: As the correlations quench the mobility of carriers, the effective pairing interaction ~ *J*/*ZW* increases due to a reduction of the quasiparticle weight *Z*^65–67^. The trend of increasing *T*_c_ is broken by a first-order SC to MI phase transition for critical *U* ~ 2 *W* which is indicated by a dotted line. Upon approaching the MI phase, the magnitude of ∣Ψ_0_∣ behaves in a dome-like shape. The *T*_c_ values of 0.8 – 1.4 × 10^−2^ *W* that we obtain from DMFT correspond to 49 – 85 K which is on the order of but quantitatively higher than the experimentally observed values. The reason for this is that we approximate the interaction to be instantaneous as well as that we neglect disorder effects and non-local correlations which reduce *T*_c_^67,68^. The same effects could mitigate the dominance of the MI phase which prevents us from observing the experimental *T*_c_ -dome^36,68^.

Turning to the correlation length, we observe that, away from *T*_c_, *ξ*(*T*) is strongly reduced to only a few lattice constants (*a* ~ 14.2 – 14.5 Å) by increasing *U*, i.e., pairing becomes very localized. At the same time, *λ*_L_(*T*) is enlarged. Hence, the condensate becomes much softer as there is a reduction of the SC stiffness ${D}_{{\rm{s}}}\propto {\lambda }_{{\rm{L}}}^{-2}$ upon increasing *U*. Fitting Eqs. (2) and (3) to our data, we obtain zero-temperature values of *ξ*_0_ = 3 – 10 nm and *λ*_L,0_ = 80 – 120 nm. Comparing our results with experimental values of *ξ*_0_ ~ 2 – 4.5 nm and *λ*_L_ ~ 200 – 800 nm^6,7,37,80,81^, we see an almost quantitative match for *ξ*_0_ and a qualitative match for *λ*_L_. Both experiment and theory consistently classify A_3_C_60_ as type II superconductors (*λ*_L_ ≫ *ξ*_0_)^37,81^.

We speculate that disorder and spontaneous orbital-symmetry breaking^74^ in the vicinity of the Mott state could lead to a further reduction of *ξ*_0_ as well as an increase of *λ*_L_ beyond what is found here for the pure system. This could bring our calculations with minimal *ξ*_0_ =3 nm closer to the experimental minimal coherence length of 2 nm revealed by measurements of large upper critical fields reaching up to a maximal *H*_c2_ = 90 T in Ref. ^80^ using *H*_c2_ = *Φ*_0_/(2*πξ*_0_) with the flux quant *Φ*_0_.

### Circumvention of the lattice BCS–BEC crossover upon boosting inverted Hund’s coupling

**Fig. 4:**
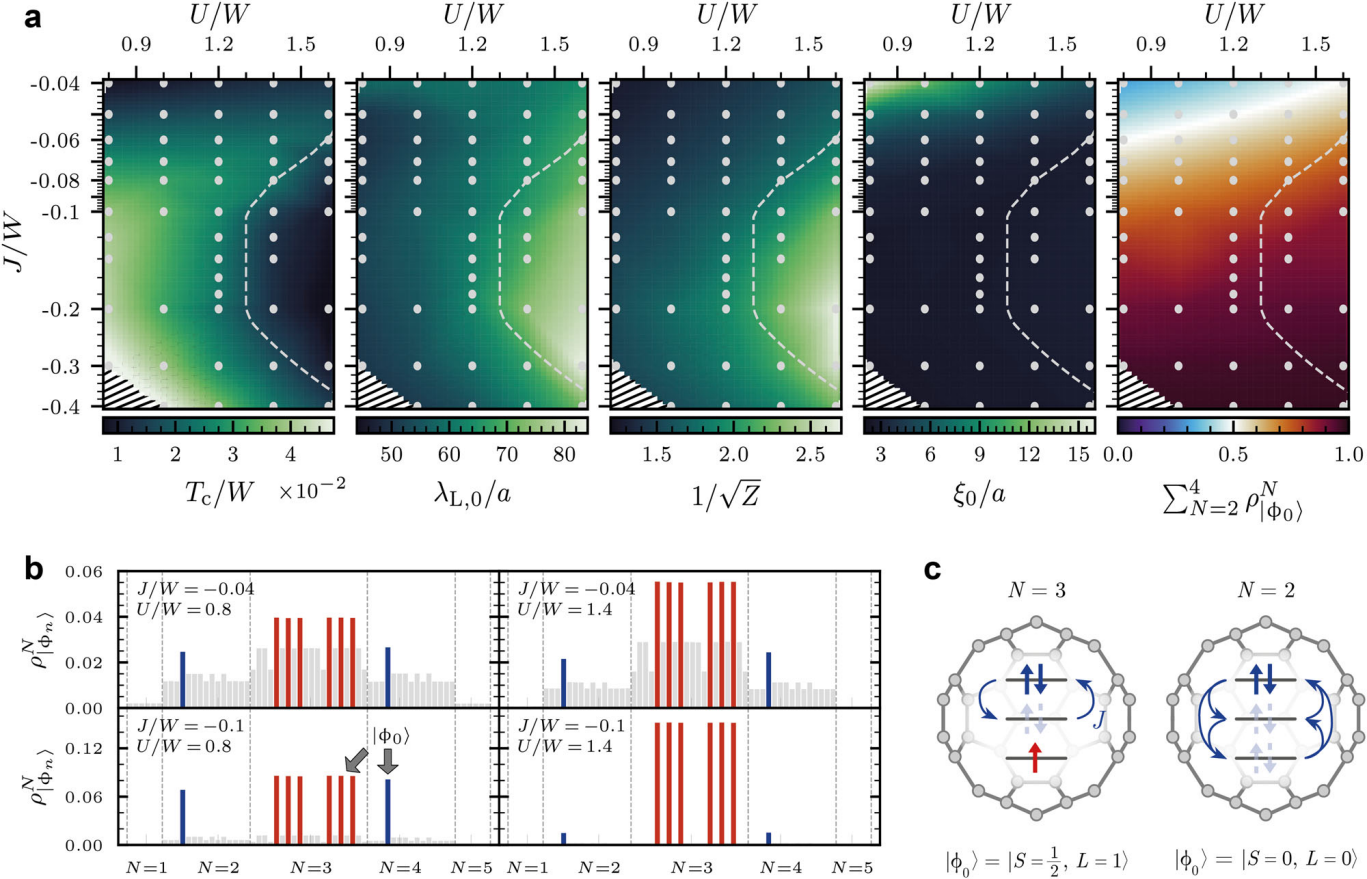
Superconducting state of the A_3_C_60_ model in the (*U*, *J*)-interaction space. **a** Critical temperature *T*_c_, zero temperature penetration depth *λ*_L,0_, inverse square root of quasiparticle weight *Z*, coherence length *ξ*_0_, and the statistical weight ${\rho }_{\left\vert {\phi }_{0}\right\rangle }^{N}$ of the local lowest energy states $\left\vert {\phi }_{0}\right\rangle$ of the *N* = 2, 3, 4 particle sectors obeying inverted Hund’s rules as a function of *U* and *J*. Gray dots show original data points used for interpolation and the dashed line indicates a region where the proximity to the Mott state leads to a suppression of the superconducting state. There is no data point at the charge degeneracy line *U* = 2∣*J*∣ in the lower left corner as marked by black ‘canceling lines. **b** Distribution of statistical weights ${\rho }_{\left\vert {\phi }_{n}\right\rangle }^{N}$ at four different interaction values *U* and *J*. (See Supplementary Note 7 for a listing of the eigenstates $\left\vert {\phi }_{n}\right\rangle$ and their respective local eigenenergies ${E}_{n}^{N}$). Red (blue) bars denote the density matrix weight of ground states in the *N* = 3 (*N* = 2, 4) particle sector, the sum of which is plotted in the last panel of (**a**). **c** Exemplary depiction of representative lowest inverted Hund’s rule eigenstates. A delocalized doublon (electron pair) fluctuates between different orbitals due to correlated pair hopping *J*.

The inverted Hund’s coupling is crucial for superconductivity in A_3_C_60_. This premise motivates us to explore in Fig. 4 the nature of the SC state in the interaction (*U*, *J*) phase space for *J* < 0 beyond ab initio estimates.

As long as ∣*J*∣ < *U*/2, we find that strengthening the negative Hund’s coupling enhances the SC critical temperature with an increase up to *T*_c_ ≈ 5 × 10^−2^ *W*, i.e., by a factor of seven compared to the ab initio motivated case of *J*/*W* = − 0.04. There is, however, a change in the role that *U* plays in the formation of superconductivity. While *U* was supportive for small magnitudes ∣*J*∣ ≲ 0.05 *W*, it increasingly becomes unfavorable for ∣*J*∣ > 0.05 *W* where *T*_c_ is reduced with increasing *U*. The effect is largest close to the MI phase where superconductivity is strongly suppressed. We indicate this proximity region by a dashed line (c.f. Supplementary Note 5-C).

**Fig. 5:**
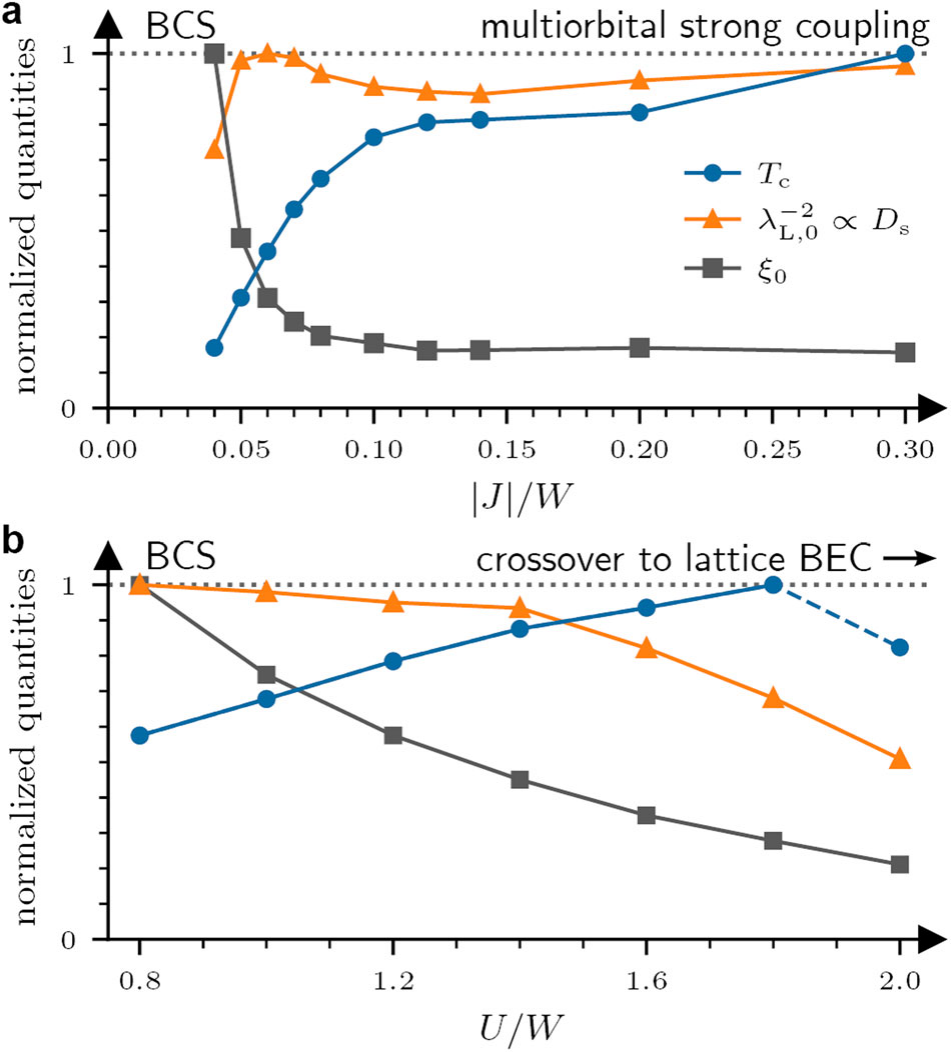
Crossover of superconducting properties between different interaction regimes in the A_3_C_60_ model. Critical temperature *T*_c_, coherence length *ξ*_0_, and stiffness ${D}_{{\rm{s}}}\propto {\lambda }_{{\rm{L}},0}^{-2}$ when approaching the multiorbital strong coupling state as function of ∣*J*∣/*W* at fixed *U*/*W* = 0.8 (**a**) and when approaching the Mott insulating state as function of *U*/*W* at fixed *J*/*W* = − 0.04 (**b**). To indicate a general trend, each quantity is normalized to its maximal value within the line cut. The phenomenology of the SC regimes resembles the BCS to lattice BEC crossover when approaching the Mott insulator (**b**), which is distinct from the multiorbital strong coupling case (**a**) (c.f. summary listed in Table 1). Note that the *T*_c_ at *U*/*W* = 2 in (**b**) corresponds to the transition from a Mott insulating state instead of a metallic phase, as visually differentiated by a dashed connecting line.

The impact of *U* on the SC state can be understood from the *U*-dependence of the London penetration depth: *λ*_L_ grows monotonously with *U* and reaches its maximum close to the MI phase. Hence, the condensate is softest in the region where Mott physics is important and it becomes stiffer at smaller *U*. We find that this fits to the behavior of the effective bandwidth *W*_eff_ = *ZW* ∝ *D*_s_ where the quasiparticle weight *Z* is suppressed upon approaching the Mott phase. The behavior of *Z* shown in Fig. 4a confirms the qualitative connection ${\lambda }_{{\rm{L}}}\propto {D}_{{\rm{s}}}^{-1/2}\propto 1/\sqrt{Z}$ for ∣*J*∣ > 0.05 *W*. The *J*-dependence of *λ*_L_ is much weaker than the *U*-dependence, as can be seen in Fig. 4a and the corresponding line-cuts in Fig. 5.

*ξ*_0_, in contrast, depends strongly on *J*. By just slightly increasing ∣*J*∣ above the ab initio estimate of ∣*J*∣, the SC state becomes strongly localized with a short coherence length on the order of 2 – 3 *a*. Remarkably, the small value of *ξ*_0_ is independent of *U* and thus the proximity to the MI phase. The localization of the condensate with *ξ*_0_ on the order of the lattice constant is reminiscent of a crossover to the BEC-type SC state. However, the dome-shaped behavior, characteristic of the lattice BCS–BEC crossover^27,29^, with decreasing *T*_c_ in the strong coupling limit is notably absent here. Instead, *T*_c_ still grows inside the plateau of minimal *ξ*_0_ when increasing the effective pairing strength proportional to ∣*J*∣ for fixed *U*/*W* (c.f. Fig. 5a). Only by diagonally traversing the (*U*, *J*) phase space, it is possible to suppress *T*_c_ inside the short *ξ*_0_ plateau with a dome structure as shown, e.g., in Ref. ^73^.

The reason for this circumvention of the lattice BCS–BEC phenomenology can be understood from an analysis of the local density matrix weights ${\rho }_{\left\vert {\phi }_{n}\right\rangle }$, where $\left\vert {\phi }_{n}\right\rangle$ refers to the eigenstates of the local Hamiltonian of our DMFT auxiliary impurity problem. We show ${\rho }_{\left\vert {\phi }_{n}\right\rangle }$ of four different points in the interaction phase space in Fig. 4b. In the region of short *ξ*_0_, the local density matrix is dominated by only eight states (red and blue bars) given by the “inverted Hund’s rule” ground states $\left\vert {\phi }_{0}\right\rangle$ of the charge sectors with *N* = 2, 3, 4 particles that are sketched in Fig. 4c. This can be seen in the last panel of Fig. 4a as the total weight of these eight states approaches one when entering the plateau of short *ξ*_0_.

By increasing ∣*J*∣, the system is driven into a strong coupling phase where local singlets are formed as Cooper pair precursors^77^ while electronic hopping is *not* inhibited. On the contrary, hopping between the *N* = 2, 3, 4 ground states is even facilitated via large negative *J*. It does so by affecting two different energy scales (c.f. Supplementary Note 7): Enhancing *J* < 0 reduces the atomic gap ${\Delta }_{{\rm{at}}}={E}_{0}^{N = 4}+{E}_{0}^{N = 2}-2{E}_{0}^{N = 3}=U-2| J|$^69^ relevant to charge excitations and thereby supports hopping. A higher negative Hund’s exchange simultaneously increases the energy *ΔE* = 2∣*J*∣ necessary to break up the orbital singlets within a fixed charge sector. As a result, unpaired electrons in the *N* = 3 state become more itinerant while the local Cooper pair binding strength increases. Since the hopping is not reduced, the SC stiffness is not compromised by larger ∣*J*∣. This two-faced role or Janus effect of negative Hund’s exchange, that localizes Cooper pairs but delocalizes electrons, can be understood as a competition of a Mott and a charge disproportionated insulator giving way for a mixed-valence metallic state in between^82^.

Correspondingly, as the superconducting state at −*J*/*W* > 0.05 relies on direct transitions between the local inverted Hund’s ground states from filling *N* = 3 to *N* = 2 and *N* = 4, the local Hubbard repulsion *U* has to fulfill two requirements for superconductivity with appreciable critical temperatures. First, significant occupation of the *N* = 2 and 4 states at half-filling requires that *U* is not too large. Otherwise, the system turns Mott insulating (around *U*/*W* ≳ 1.6 for enhanced ∣*J*∣) and the SC phase stiffness is reduced upon increasing *U* towards the Mott limit. At the same time, a significant amount of statistical weight of the *N* = 3 states demands that *U* must not be too small either. We find that *Δ*_at_ > 0 and thus *U* > 2∣*J*∣ is necessary for robust SC pairing. At *Δ*_at_ < 0 there is a predominance of *N* = 2 and 4 states, which couple kinetically only in second-order processes and which are susceptible to charge disproportionation^82^. Our analysis shows that a sweet spot for a robust SC state with high phase stiffness and large *T*_c_ exists for ∣*J*∣ approaching *U*/2.

## Discussion

We summarize the overall change from a weak-coupling BCS state to the multiorbital strong coupling SC state characterized by *T*_c_, *ξ*_0_, and ${D}_{{\rm{s}}}\propto {\lambda }_{{\rm{L}}}^{-2}$ upon enhancing ∣*J*∣ in Fig. 5a and contrast it with the lattice BCS–BEC phenomenology in Table 1. Behavior as in the lattice BCS–BEC crossover can be found upon approaching the MI state (Fig. 5b), where strong local repulsion *U* decreases *ξ*_0_ and superconductivity compromises stiffness. As discussed above, we cannot observe a proper *T*_c_ dome that is seen in the experimental phase diagram due to the MI state dominating over the SC state in our calculations for fixed *J*/*W* = − 0.04^36,67,68^. However, an additional analysis of the SC gap *Δ* and coupling strength in Supplementary Note 6 indicates that our calculations are indeed in the vicinity of the crossover region. Diagonally traversing the (*U*, *J*) interaction plane by increasing *U* for *J* = − *γU* (*γ* ≤ 0.5) leads to a similar conclusion, although a *T*_c_ dome can emerge more easily^73^ as charge excitations controlled by *Δ*_at_ are suppressed at a slower pace.

**Table 1:** Characteristics of the BCS, crossover to lattice BEC, and multiorbital strong coupling limits

	BCS limit	Crossover to lattice BEC limit	Multiorbital strong coupling
***T***_**c**_	Increase with pairing interaction, *T*_c_ ∝ *Δ*(*T* = 0)	Decrease with pairing interaction, *T*_c_ ∝ *D*_s_	Increase with pairing interaction, *T*_c_ ∝ *Δ*, *D*_s_
***ξ***_0_	Long (*ξ*_0_ ≫ *a*)	Short (*ξ*_0_ ≳ *a*), increasing in (deep) BEC limit	Short (*ξ*_0_ ~ *a*), constant with pairing interaction
${{\boldsymbol{D}}}_{{\bf{s}}}{{\propto }}{{\boldsymbol{\lambda }}}_{{\bf{L}}}^{-{\bf{2}}}$	Constant with pairing interaction	Decreasing with pairing interaction	Increasing/constant with pairing interaction
***Δ***/***E***_**F**_	Small value (*Δ*/*E*_F_ ≪ 1)	Large value (*Δ*/*E*_F_ ≳ 1)	Intermediate value ($\Delta /{E}_{{\rm{F}}} \sim {\mathcal{O}}(0.1)$)
**Corresponding** (***U***, ***J***) **region**	~ small *U*/*W* ~ small ∣*J*∣/*W*	Onset at ~large *U*/*W*	~ small to intermediate *U*/*W* ~ large ∣*J*∣/*W*, ∣*J*∣/*U* < 1/2

Behavior of the critical temperature *T*_c_, coherence length *ξ*_0_, superconducting stiffness *D*_s_, and the ratio of superconducting gap *Δ* to the Fermi energy *E*_F_ (coupling strength) in the BCS, crossover to lattice BEC^24^ and “multiorbital strong coupling” limit. We indicate in the last row where we can find the respective behavior in the interaction plane of the A_3_C_60_ model. We do not observe the deep BEC limit for large *U*/*W* and small *J*/*W*, but signatures indicating the onset of the BCS–BEC crossover (c.f. Figs. 4a and 5 as well as Supplementary Note 6).

Thus, two distinct localized SC states exist in the multiorbital model A_3_C_60_ – one facilitated by local Hubbard repulsion *U* and the other by enhanced inverted Hund’s coupling ∣*J*∣. One might speculate under which conditions THz driving or more generally photoexcitation^15,17,18,38^ could enhance ∣*J*∣ and steer A_3_C_60_ into this high-*T*_c_ and short-*ξ*_0_ strong coupling region, e.g., via quasiparticle trapping or displacesive meta-stability^83^. Further experimental characterization via observables susceptible to changes in *ξ*_0_ and *λ*_L_, like critical fields, currents (see Supplementary Note 1-B), or thermoelectric and thermal transport coefficients^8,14^, can lash down the possibility of this scenario. Twisted bilayer graphene might be another platform to host the mechanism proposed in this work as an inverted Hund’s pairing similar to that in A_3_C_60_ is currently under discussion^84–86^.

On general grounds, the bypassing of the usual lattice BCS–BEC scenario via multiorbital physics is promising for optimization of superconducting materials to achieve higher critical currents or temperatures. Generally, limits of accessible *T*_c_ are unknown with so far only a rigorous boundary existing for two-dimensional (2D) systems^87,88^. An empirical upper bound to *T*_c_ emerges from the Uemura classification^5–8^ which compares *T*_c_ to the Fermi temperature *T*_F_ = *E*_F_/*k*_B_ ∝ *D*_s_: the temperature ${T}_{{\rm{BEC}}}^{3{\rm{D}}}={T}_{{\rm{F}}}/4.6$ of a three-dimensional (3D) non-interacting BEC. Most superconducting materials, including cuprate materials with the highest known *T*_c_ at ambient conditions, however, are not even close to this boundary; typically, *T*_c_/*T*_BEC_ = 0.1 – 0.2 for unconventional superconductors. Notable exceptions are monolayer FeSe^89^ (*T*_c_/*T*_BEC_ = 0.43), twisted graphitic systems^26,84^ (*T*_c_/*T*_BEC_ ~ 0.37 – 0.57), and 2D semiconductors at low carrier densities^25^ (*T*_c_/*T*_BEC_ ~ 0.36 – 0.56) which all reach close to the 2D boundary ${T}_{{\rm{c,lim}}}^{2{\rm{D}}}/{T}_{{\rm{BEC}}}^{3{\rm{D}}}\approx 0.575$^87^. Only ultracold Fermi gases can be tuned very close to the optimal *T*_BEC_^23,24^.

The empirical limitations of *T*_c_ in most unconventional superconductors with rather high densities make sense from the standpoint and constraints of the lattice BCS–BEC crossover. In this picture, high densities seem favorable for reaching high *T*_c_ as electrons can reach high intrinsic energy scales. Yet, lattice effects and their negative impact on the kinetic energy of superconducting carriers become also more pronounced, preventing *T*_c_ to reach *T*_BEC_. Possible routes to evade these constraints include quantum geometric and hybridization related band structure effects^90–98^ as well as the multiorbital Hund’s interaction effects triggering substantial localized stiffness uncovered here.

We emphasize that the framework to calculate the coherence length *ξ*_0_ and the London penetration depth *λ*_L_ introduced in this work can be implemented in any Green function or density functional based approach to superconductivity without significant increase of the numerical complexity. Thus, our work opens the gate for “in silico” superconducting materials’ optimization targeting not only *T*_c_ but also *ξ*_0_ and *λ*_L_. On a more fundamental level, availability of *ξ*_0_ and *λ*_L_ rather than *T*_c_ alone can provide more constraints on possible pairing mechanisms through more rigorous theory-experiment comparisons, particularly in the domain of superconductors with strong electronic correlations.

## Methods

### Dynamical mean-field theory under the constraint of finite-momentum pairing

We study the multiorbital interacting model *H*_kin_ + *H*_int_ of A_3_C_60_ using Dynamical Mean-Field theory (DMFT) in Nambu space under the constraint of finite-momentum pairing (FMP). In DMFT, the lattice model is mapped onto a single Anderson impurity problem with a self-consistent electronic bath that can be solved numerically exactly. The self-energy becomes purely local *Σ*_*ij*_(*iω*_*n*_) = *δ*_*ij*_*Σ*(*iω*_*n*_) capturing all local correlation effects. To this end, we have to solve the following set of self-consistent equations

9 $$\left\{ \begin{array}{ll} {\underline{G}}_{{\mathrm{loc}}}(i\omega_n) &= \frac{1}{N_{{\boldsymbol{k}}}} \sum\limits_{{\boldsymbol{k}}} {\underline{G}}_{{\boldsymbol{k}}}(i\omega_n)\\ &= \frac{1}{N_{{\boldsymbol{k}}}} \sum\limits_{{\boldsymbol{k}}} \left[(i\omega_n + \mu){\mathbb{1}} - {\underline{h}}({\boldsymbol{k}}) - {\underline{{\Sigma}}}(i\omega_n) \right]^{-1}\,\\ {\underline{G}}_{{\mathrm{W}}}^{-1}(i\omega_n) &= {\underline{G}}^{-1}_{{\mathrm{loc}}}(i\omega_n) + {\underline{{\Sigma}}}(i\omega_n)\,\\ {\underline{{\Sigma}}}(i\omega_n) &= {\underline{G}}_{{\mathrm{W}}}^{-1}(i\omega_n) - {\underline{G}}_{{\mathrm{imp}}}^{-1}(\omega_n) \end{array}\right.$$

in DMFT^51^. Here, the local Green function *G*_loc_ is obtained from the lattice Green function *G*_***k***_ (first line) in order to construct the impurity problem by calculating the Weiss field *G*_W_ (second line). Solving the impurity problem yields the impurity Green function *G*_imp_ from which the self-energy *Σ* is derived (third line). Underlined quantities denote matrices with respect to orbital indices (*α*), ${\mathbb{1}}$ is the unit matrix in orbital space, *ω*_*n*_ = (2*n* + 1)*πT* denote Matsubara frequencies, ${h}_{\alpha \gamma }({\boldsymbol{k}})={\sum }_{j}{t}_{\alpha \gamma }({{\boldsymbol{R}}}_{j}){{\rm{e}}}^{i{\boldsymbol{k}}{{\boldsymbol{R}}}_{j}}$ is the Fourier transform of the hopping matrix in Eq. (8), *μ* indicates the chemical potential, and *N*_***k***_ is the number of ***k***-points in the momentum mesh. Convergence of the self-consistency problem is reached when the equality ${\underline{G}}_{{\rm{loc}}}(i{\omega }_{n})={\underline{G}}_{{\rm{imp}}}(i{\omega }_{n})$ holds.

We can study superconductivity directly in the symmetry-broken phase by extending the formalism to Nambu–Gor’kov space. The Nambu–Gor’kov function under the constraint of FMP (c.f. Eq. (4)) on Matsubara frequencies

10 $${[{\underline{\mathcal{G}}}_{{\boldsymbol{q}}}(i\omega_n,{\boldsymbol{k}})]}^{-1} =\left(\begin{array}{rc} (i\omega_n + \mu){\mathbb{1}} - {\underline{h}}({\boldsymbol{k}}+\frac{{\boldsymbol{q}}}{2}) - {\underline{{\Sigma}}}^{{\mathrm{N}}}(i\omega_n) &-{\underline{{\Sigma}}}^{{\mathrm{AN}}}(i\omega_n)\\ -{\underline{{\Sigma}}}^{{\mathrm{AN}}}(i\omega_n) &(i\omega_n - \mu){\mathbb{1}} + {\underline{h}}(-{\boldsymbol{k}}+\frac{{\boldsymbol{q}}}{2}) + [{\underline{{\Sigma}}}^{{\mathrm{N}}}]^*(i\omega_n) \end{array}\right)$$

then takes the place of the lattice Green function ${\underline{G}}_{{\boldsymbol{k}}}(i{\omega }_{n})\mapsto {\underline{{\mathcal{G}}}}_{{\boldsymbol{q}}}(i{\omega }_{n},{\boldsymbol{k}})$ in the self-consistency cycle in Eq. (9). In addition to the normal component *Σ*^N^ ≡ *Σ*, the self-energy gains an anomalous matrix element *Σ*^AN^ for which the gauge is chosen such that it is real-valued.

We use a 35 × 35 × 35 ***k***-mesh and 43200 Matsubara frequencies to set up the lattice Green function in the DMFT loop. In order to solve the local impurity problem, we use a continuous-time quantum Monte Carlo (CT-QMC) solver^99^ based on the strong coupling expansion in the hybridization function (CT-HYB)^100^. Details on the implementation can be found in Refs. ^67,68^. Depending on calculation parameters (*T*, *U*, *J*) and proximity to the superconducting transition, we perform between 2.4 × 10^6^ up to 19.2 × 10^6^ Monte Carlo sweeps and use a Legendre expansion with 50 up to 80 basis functions. Some calculations very close to the onset of the superconducting phase transition (depending on *T* or ***q***) needed >200 DMFT iterations until convergence. We average 10 or more converged DMFT iterations and calculate the mean value and standard deviation in order to estimate the uncertainty of the order parameter originating from the statistical noise of the QMC simulation.

Further details on code implementation are given in Supplementary Note 4. It entails a comparison of finite-momentum pairing to the zero-momentum case^51^ in the Nambu–Gor’kov formalism, an explanation on the readjustment of the chemical potential *μ* to fix the electron filling, and a simplification of the Matsubara sum for calculating ***j***_***q***_ in Eq. (6).

## Supplementary information

Supplementary Information

## Data Availability

The data that support the findings of this study are available from the corresponding author upon reasonable request.
