# Supplementary material for: Bypassing the lattice BCS–BEC crossover in strongly correlated superconductors through multiorbital physics
Source: NPJ Quantum Mater. 2024 Dec 10;9(1):100. doi: 10.1038/s41535-024-00706-7 (PMC11631768; doi:10.1038/s41535-024-00706-7)
Supplement: Supplementary file 1 — Supplementary Information [file 41535_2024_706_MOESM1_ESM.pdf]

Supplementary Information:

Bypassing the lattice BCS–BEC crossover in strongly correlated superconductors through multiorbital physics

Niklas Witt*, Yusuke Nomura, Sergey Brener, Ryotaro Arita, Alexander I. Lichtenstein, Tim O. Wehling

*Corresponding author: niklas.witt@physik.uni-hamburg.de

Contents

1 Phenomenological Ginzburg–Landau theory with finite-momentum pairing 1
- 1-A Order parameter and supercurrent density . . . 1
- 1-B Relation to experimental observables . . . 4

2 Generalized Bloch theorem in Nambu space 4

3 Derivation of the supercurrent density 6

4 Numerical implementation of DMFT with FMP constraint 8
- 4-A Nambu–Gor'kov formalism with finite-momentum pairing . . . 8
- 4-B Determination of chemical potential . . . 11
- 4-C Handling the Matsubara summation in the calculation of the current density . . 11
- 4-D Lattice model details and current direction . . . 13

5 Details on the calculation of $|\Psi_q|$, ξ_0, j_{dp}, and λ_{L} from DMFT 15
- 5-A Order parameter and coherence length . . . 15
- 5-B Current density and penetration depth . . . 19
- 5-C Proximity region to the Mott transition . . . 20

6 Analysis of superconducting gap and coupling strength 21

7 Atomic limit of three-orbital model with inverted Hund's coupling 24

Supplementary Note 1: Phenomenological Ginzburg–Landau theory with finite-momentum pairing

The Ginzburg–Landau (GL) framework is a phenomenological (macroscopic) approach to the superconducting phase transition. Here, we illustrate with GL theory how introducing a FMP constraint gives access to the London penetration depth λ_L, coherence length ξ_0, and also the depairing current j_dp. The GL description with FMP has been discussed in other contexts like the superconducting diode effect [55] and Fulde–Ferrel–Larkin–Ovchinnikov (FFLO) theory [59]. Note that, as mentioned in the main text, we use the term 'FMP' to refer exclusively to the order parameter with a helical phase variation and constant amplitude, whereas it is sometimes also used in the context of pair density waves [58, 63] which imprint an amplitude modulation on the superconducting gap.

1-A Order parameter and supercurrent density

We start from the GL expansion of the free energy of the symmetry-broken state in terms of the complex superconducting order parameter (OP) $\Psi(\boldsymbol{r}) = |\Psi(\boldsymbol{r})|\mathrm{e}^{i\varphi(\boldsymbol{r})}$ close to the phase transition point T_c which reads

$$\mathcal{F}[\Psi] = \mathcal{F}_\mathrm{N} + \int \mathrm{d}^3r \left[\alpha(T)|\Psi(\boldsymbol{r})|^2 + \frac{b}{2}|\Psi(\boldsymbol{r})|^4 + \frac{\hbar^2}{2m^*}|\nabla\Psi(\boldsymbol{r})|^2\right] \tag{1}$$

where $\mathcal{F}_\mathrm{N}$ is the free energy of the normal state and $\alpha(T) = \alpha_0(T - T_\mathrm{c})$ ($\alpha_0 > 0$), $b > 0$, and m^* are the material dependent GL parameters. The GL functional encodes the two types of collective modes that emerge in the symmetry-broken state: fluctuations of the amplitude (Higgs mode) and the phase (Nambu–Goldstone mode) of the OP. The constraint of FMP means that we require the Cooper pairs to carry a finite fixed momentum $\boldsymbol{q}$, which translates to the requirement for the OP to be of the form $\Psi_{\boldsymbol{q}}(\boldsymbol{r}) = |\Psi_{\boldsymbol{q}}|\mathrm{e}^{i\boldsymbol{q}\boldsymbol{r}}$. Then, the GL free energy density becomes

$$f_\mathrm{GL}[\Psi_{\boldsymbol{q}}] = (\mathcal{F}[\Psi_{\boldsymbol{q}}] - \mathcal{F}_\mathrm{N})/V = \alpha|\Psi_{\boldsymbol{q}}|^2 + \frac{b}{2}|\Psi_{\boldsymbol{q}}|^4 + \frac{\hbar^2 q^2}{2m^*}|\Psi_{\boldsymbol{q}}|^2 \tag{2}$$

The gradient term in this expression has an associated length scale which is the temperature-dependent correlation length $\xi(T)$ given by

$$\xi(T) = \sqrt{\frac{\hbar^2}{2m^*|\alpha|}} = \xi_0\left(1 - \frac{T}{T_\mathrm{c}}\right)^{-\frac{1}{2}} \tag{3}$$

with the coherence length $\xi_0 = \hbar/\sqrt{\alpha_0 m^* T_\mathrm{c}}$ at $T = 0$ [4]. The system's stationary point is calculated from

$$\frac{\delta f_\mathrm{GL}}{\delta\Psi^*_{\boldsymbol{q}}} = 2\Psi_{\boldsymbol{q}}\left[\alpha(1 - \xi^2 q^2) + b|\Psi_{\boldsymbol{q}}|^2\right] \stackrel{!}{=} 0 \tag{4}$$

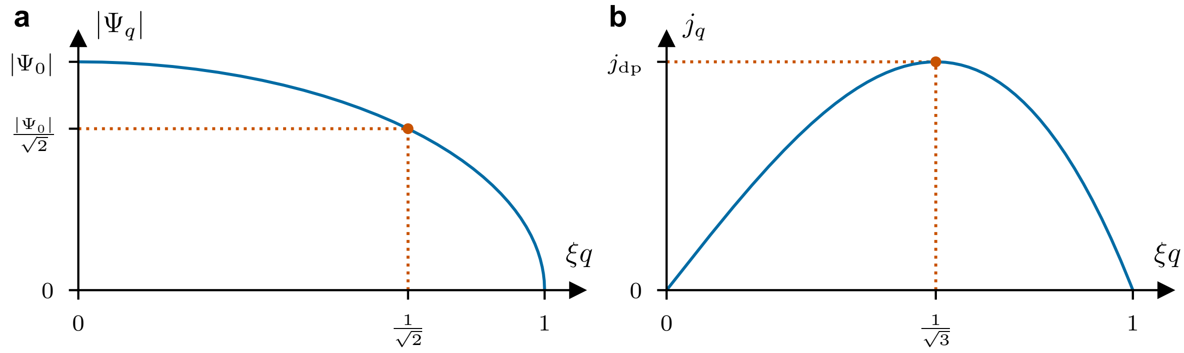

Supplementary Figure 1 | Ginzburg–Landau solution for finite-momentum pairing. Ginzburg–Landau expectation of the momentum (q)-dependent (**a**) order parameter modulus $|\Psi_q|$ and (**b**) the concomitant supercurrent density j_q of a system under the constraint of finite-momentum pairing superconductivity in units of the correlation length ξ. We marked the characteristic length scale on which the order parameter is reduced and the point of the depairing current density j_{dp}.

which results in the $\boldsymbol{q}$-dependence of the OP given by

$$|\Psi_{\boldsymbol{q}}(T)|^2 = |\Psi_0(T)|^2(1 - \xi(T)^2 q^2) \tag{5}$$

with the homogeneous OP $|\Psi_0(T)|^2 = -\alpha(T)/b \propto T - T_{\mathrm{c}}$. We plot this relation in Figure 1a. It shows that the OP amplitude is reduced compared to the zero-momentum pairing case for any finite $q = |\boldsymbol{q}| > 0$. This suppression is induced by the nonlinear coupling of the Higgs mode to the phase mode [3]. For some critical momentum value q_{c}, superconducting order breaks down completely ($\Psi_{q_{\mathrm{c}}} = 0$) because the kinetic energy from phase modulations exceeds the gain in energy from pairing. In GL theory, this value is given exactly by $q_{\mathrm{c}} = \xi(T)^{-1}$ (c.f. Eq. (5)). The temperature dependence of the OP and extracted $\xi(T)$ gives access to the coherence length ξ_0 via Eq. (3) (c.f. Note 5-A).

The $\boldsymbol{q}$-dependence of the OP also connects to the depairing current j_{dp} [61]. To see this, we derive the current density in the superconducting state. For this purpose, we (briefly) introduce a vector potential $\boldsymbol{A}$ via minimal coupling to the free energy [4, 55]

$$\begin{aligned} f_{\mathrm{GL}}[\Psi_{\boldsymbol{q}}] &= \alpha|\Psi_{\boldsymbol{q}}|^2 + \frac{b}{2}|\Psi_{\boldsymbol{q}}|^4 + \frac{1}{2m^*}|(i\hbar\nabla + e^*\boldsymbol{A})\Psi_{\boldsymbol{q}}(\boldsymbol{r})|^2 \\ &= \alpha|\Psi_{\boldsymbol{q}}|^2 + \frac{b}{2}|\Psi_{\boldsymbol{q}}|^4 + \frac{1}{2m^*}|(\hbar^2 q^2 - 4\hbar e\boldsymbol{q}\cdot\boldsymbol{A} + 4e^2A^2)|\Psi_{\boldsymbol{q}}|^2 \end{aligned} \tag{6}$$

where we choose the Coulomb-gauge $\nabla \cdot \boldsymbol{A} = 0$ and use $e^* = 2e$. We can explicitly set e^* and, in this regard, treat the charge differently to the mass m^* because e^* and $(e^*)^2$ couple the same way to the OP, i.e., the charge of the Cooper pair is not renormalized. We obtain the steady-state

current density from the first derivative with respect to the vector potential

$$\boldsymbol{j} = -\frac{\delta f_{\rm GL}}{\delta \boldsymbol{A}}\bigg|_{\boldsymbol{A}=0} = \frac{2\hbar e}{m^*}|\Psi_{\boldsymbol{q}}|^2\left(\boldsymbol{q} - \frac{2\pi}{\Phi_0}\boldsymbol{A}\right)\bigg|_{\boldsymbol{A}=0} = \frac{2\hbar e}{m^*}|\Psi_{\boldsymbol{q}}|^2\boldsymbol{q} \tag{7}$$

with the magnetic flux quantum $\Phi_0 = h/(2e) = \pi\hbar/e$. By inserting the OP from Eq. (5), we obtain a $\boldsymbol{q}$-dependent expression for the current density (T-dependence suppressed)

$$\boldsymbol{j}_{\boldsymbol{q}} = \frac{2\hbar e}{m^*}|\Psi_0|^2(1-\xi^2q^2)q\,\hat{\boldsymbol{q}} \tag{8}$$

that directly shows how the Cooper pairs carry the supercurrent with their finite center-of-mass momentum along the direction $\hat{\boldsymbol{q}} = \boldsymbol{q}/q$. The current density, $j_q = |\boldsymbol{j}_q|$, is a non-monotonous function of q that exhibits a maximum called depairing current, $j_{\rm dp}$ (c.f. Figure 1b). $j_{\rm dp}$ can be explicitly calculated from $\partial j_q/\partial q = 0$ which yields $q_{\max} = 1/(\sqrt{3}\xi)$ and

$$j_{\rm dp} \equiv j_{q_{\max}} = \frac{4}{3\sqrt{3}}\frac{e\hbar|\Psi_0|^2}{m^*\xi} \tag{9}$$

Since the supercurrent $\boldsymbol{j}$ is directly related to the vector potential $\boldsymbol{A}$, it is possible to derive the London equation within GL theory. We obtain the second London equation by taking the curl of Eq. (7) with $\boldsymbol{q} = 0$ such that

$$\frac{1}{\mu_0\lambda_{\rm L}^2}\boldsymbol{B} = -\nabla\times\boldsymbol{j} = \frac{4\pi\hbar e|\Psi_0|^2}{m^*\Phi_0}\nabla\times\boldsymbol{A} = \frac{4e^2|\Psi_0|^2}{m^*}\boldsymbol{B} \tag{10}$$

Here, the London penetration depth $\lambda_{\rm L}$ is introduced which, in turn, can be reformulated to depend on the correlation length and depairing current:

$$\lambda_{\rm L}(T) = \sqrt{\frac{m^*}{4\mu_0 e^2|\Psi_0(T)|^2}} \overset{\text{Eq. (9)}}{=} \sqrt{\frac{\Phi_0}{3\sqrt{3}\pi\mu_0\xi(T)j_{\rm dp}(T)}} = \lambda_{\rm L,0}\left(1-\left(\frac{T}{T_{\rm c}}\right)^4\right)^{-\frac{1}{2}} \tag{11}$$

The temperature dependence with the quartic power stated here is empirical (derived from the Gorter–Casimir model) and often used to fit experimental data [39, 40]. In Note 5-B, we show that this temperature dependence models our DMFT data better than a linear power law as assumed in GL theory.

To summarize, we obtain the correlation length $\xi(T)$ and depairing current $j_{\rm dp}(T)$ from analyzing the OP $\Psi_{\boldsymbol{q}}(\boldsymbol{r}) = |\Psi_{\boldsymbol{q}}|\mathrm{e}^{i\boldsymbol{q}\boldsymbol{r}}$ subject to the FMP constraint. In a second step, we can derive the London penetration depth $\lambda_{\rm L}(T)$ from these two quantities. This connection also holds in microscopic theories, where we calculate the OP and current density from the Nambu–Gor'kov Green function (see main text and Note 5). Lastly, we want to note that the analysis of length scales $\xi(T)$ and $\lambda_{\rm L}(T)$ as done in this work is equivalent to discussing energy scales of Higgs and Nambu–Goldstone modes [3].

1-B Relation to experimental observables

ξ, λ_L, and j_dp link to several experimental observables [40, 61] where we suppress the T-dependence for brevity. j_dp constitutes an upper theoretical bound to the critical current density, j_c, that limits the maximal current which the superconducting state of a material can endure and which is the observable measured in experiment. The value of j_c crucially depends on sample geometry and defect densities as a current only flows near the surface shell of thickness $\sim \lambda_\mathrm{L}$.

ξ and λ_L are used to distinguish type I ($\xi/\lambda_\mathrm{L} > \sqrt{2}$) and type II ($\xi/\lambda_\mathrm{L} < \sqrt{2}$) superconductors and they relate to the critical magnetic fields: The first critical magnetic field

$$H_\mathrm{c1} = \frac{\Phi_0}{4\pi\mu_0\lambda_\mathrm{L}^2} \ln \frac{\lambda_\mathrm{L}}{\xi} \tag{12}$$

that separates the Meissner and Abrikosov vortex lattice phases, the second critical magnetic field

$$H_\mathrm{c2} = \frac{\Phi_0}{2\pi\mu_0\xi^2} \tag{13}$$

that determines the magnetic field strength boundary at which the superconductor becomes a normal metal, and the thermodynamic critical field

$$H_\mathrm{c,th} = \frac{\Phi_0}{2\sqrt{2}\pi\mu_0\xi\lambda} = \sqrt{\frac{\alpha^2}{b\mu_0}} = \sqrt{\frac{2}{\mu_0}(f_\mathrm{N} - f_\mathrm{SC})|_{\mathrm{min},q=0}} \tag{14}$$

Supplementary Note 2: Generalized Bloch theorem in Nambu space

Crystal momentum $\boldsymbol{k}$ is in general not a good quantum number for systems with spatial inhomogeneity. This applies to the situation of an arbitrary spatially varying superconducting gap $\Delta(\boldsymbol{r}) = |\Delta(\boldsymbol{r})|\mathrm{e}^{i\varphi(\boldsymbol{r})}$. In the special case of FMP with an OP of Fulde–Ferrel-type [57], i.e., with helical phase $\varphi(\boldsymbol{r}) = \boldsymbol{q}\cdot\boldsymbol{r}$ and constant amplitude $|\Delta(\boldsymbol{r})| = |\Delta|$, however, a generalized lattice translation symmetry exists in Nambu space that implies a generalized Bloch theorem: We define a generalized translation operator $\mathcal{T}_n$ that acts on the Nambu spinor $\underline{\psi}^\dagger = \begin{pmatrix}\psi_\uparrow^\dagger, & \psi_\downarrow\end{pmatrix}$ of field operators $\psi^{(\dagger)}(\boldsymbol{r})$:

$$\mathcal{T}_n\underline{\psi}(\boldsymbol{r}) = \mathcal{T}_n\begin{pmatrix}\psi_\uparrow(\boldsymbol{r})\\ \psi_\downarrow^\dagger(\boldsymbol{r})\end{pmatrix} = \begin{pmatrix}\mathrm{e}^{i\phi_n/2}\ \psi_\uparrow(\boldsymbol{r}+\boldsymbol{R}_n)\\ \mathrm{e}^{-i\phi_n/2}\ \psi_\downarrow^\dagger(\boldsymbol{r}+\boldsymbol{R}_n)\end{pmatrix} = \mathrm{e}^{i\phi_n\sigma_z/2}\,\underline{\psi}(\boldsymbol{r}+\boldsymbol{R}_n) \tag{15}$$

Here, the spinor is not only shifted by a Bravais lattice vector $\boldsymbol{R}_n$ but it is also rotated by the angle $\phi_n = \boldsymbol{q}\cdot\boldsymbol{R}_n$ about the z-axis on the Bloch sphere with σ_z being a Pauli matrix.

In the following, we show that the translation defined by Eq. (15) leaves the Hamiltonian $H = H_0 + H_{\rm SC}$ of a superconducting system consisting of lattice (H_0) and pairing ($H_{\rm SC}$) term invariant, i.e., that it obeys a generalized Bloch theorem. In Nambu space, the Hamiltonian takes in d dimensions the form

$$\begin{aligned} H_0 + H_{\rm SC} &= \int \mathrm{d}^d r \left[\sum_\sigma \left\{ h(\boldsymbol{r}) \psi^\dagger_\sigma(\boldsymbol{r}) \psi_\sigma(\boldsymbol{r}) \right\} + \Delta_{\boldsymbol{q}}(\boldsymbol{r}) \psi_\downarrow(\boldsymbol{r}) \psi_\uparrow(\boldsymbol{r}) + \Delta^*_{\boldsymbol{q}}(\boldsymbol{r}) \psi^\dagger_\uparrow(\boldsymbol{r}) \psi^\dagger_\downarrow(\boldsymbol{r}) \right] \\ &= \int \mathrm{d}^d r \left[\underline{\psi}^\dagger(\boldsymbol{r}) \begin{pmatrix} h(\boldsymbol{r}) & \Delta^*_{\boldsymbol{q}}(\boldsymbol{r}) \\ \Delta_{\boldsymbol{q}}(\boldsymbol{r}) & -h(\boldsymbol{r}) \end{pmatrix} \underline{\psi}(\boldsymbol{r}) \right] \\ &= \int \mathrm{d}^d r \ \left[\underline{\psi}^\dagger(\boldsymbol{r}) \left(h(\boldsymbol{r}) \sigma_z + \mathrm{Re}\left\{ \Delta_{\boldsymbol{q}}(\boldsymbol{r}) \right\} \sigma_x + \mathrm{Im}\left\{ \Delta_{\boldsymbol{q}}(\boldsymbol{r}) \right\} \sigma_y \right) \underline{\psi}(\boldsymbol{r}) \right] \end{aligned} \tag{16}$$

with the single-particle Hamiltonian $h(\boldsymbol{r}) = -\frac{\hbar^2}{2m}\nabla^2 + V(\boldsymbol{r})$ containing the lattice periodic potential $V(\boldsymbol{r}) = V(\boldsymbol{r} + \boldsymbol{R}_n)$ and with the FMP pairing potential or gap function $\Delta_{\boldsymbol{q}}(\boldsymbol{r}) = |\Delta| \mathrm{e}^{i\boldsymbol{q}\cdot\boldsymbol{r}}$. Since $\Delta \propto \Psi$, the superconducting gap carries over the phase dependence of the order parameter under the FMP constraint. From the last line of Eq. (16), it is immediately clear that H_0 is invariant under translation $\mathcal{T}_n$ in Nambu space, as $\mathcal{T}_n$ trivially commutes with $h(\boldsymbol{r})\sigma_z$. The invariance of $H_{\rm SC}$ follows from the phase shift of the pairing field $\Delta_{\boldsymbol{q}}(\boldsymbol{r} + \boldsymbol{R}_n) = \Delta_{\boldsymbol{q}}(\boldsymbol{r}) \mathrm{e}^{i\phi_n}$ associated with

$$\begin{aligned} H_{\rm SC} &= \int \mathrm{d}^d r \left[\underline{\psi}^\dagger(\boldsymbol{r}) \begin{pmatrix} 0 & \Delta^*_{\boldsymbol{q}}(\boldsymbol{r}) \\ \Delta_{\boldsymbol{q}}(\boldsymbol{r}) & 0 \end{pmatrix} \underline{\psi}(\boldsymbol{r}) \right] \\ &= \int \mathrm{d}^d r \left[\underline{\psi}^\dagger(\boldsymbol{r} + \boldsymbol{R}_n) \begin{pmatrix} 0 & \Delta^*_{\boldsymbol{q}}(\boldsymbol{r} + \boldsymbol{R}_n) \\ \Delta_{\boldsymbol{q}}(\boldsymbol{r} + \boldsymbol{R}_n) & 0 \end{pmatrix} \underline{\psi}(\boldsymbol{r} + \boldsymbol{R}_n) \right] \\ &= \int \mathrm{d}^d r \left[\underline{\psi}^\dagger(\boldsymbol{r} + \boldsymbol{R}_n) \begin{pmatrix} 0 & \Delta^*_{\boldsymbol{q}}(\boldsymbol{r}) \mathrm{e}^{-i\phi_n} \\ \Delta_{\boldsymbol{q}}(\boldsymbol{r}) \mathrm{e}^{i\phi_n} & 0 \end{pmatrix} \underline{\psi}(\boldsymbol{r} + \boldsymbol{R}_n) \right] \\ &= \int \mathrm{d}^d r \left[\underline{\psi}^\dagger(\boldsymbol{r} + \boldsymbol{R}_n)\, \mathrm{e}^{-i\phi_n\sigma_z/2} \begin{pmatrix} 0 & \Delta^*_{\boldsymbol{q}}(\boldsymbol{r}) \\ \Delta_{\boldsymbol{q}}(\boldsymbol{r}) & 0 \end{pmatrix} \mathrm{e}^{i\phi_n\sigma_z/2} \underline{\psi}(\boldsymbol{r} + \boldsymbol{R}_n) \right] \\ &= \int \mathrm{d}^d r \left[\underline{\psi}^\dagger(\boldsymbol{r}) \mathcal{T}_n^\dagger \begin{pmatrix} 0 & \Delta^*_{\boldsymbol{q}}(\boldsymbol{r}) \\ \Delta_{\boldsymbol{q}}(\boldsymbol{r}) & 0 \end{pmatrix} \mathcal{T}_n \underline{\psi}(\boldsymbol{r}) \right] \end{aligned}$$

Thus, the generalized translation in Eq. (15) is a symmetry of the system and (generalized) crystal momentum $\boldsymbol{k}$ constitutes a good quantum number in the case of FMP superconductivity. Note, though, that this is not true for pair density waves or generally speaking more complex FFLO-type pairings which also modulate the amplitude of the OP. In this case, methods employing supercells to accommodate for the extent of the OP modulation are necessary as was done, e.g., in Refs. [101, 102].

Supplementary Note 3: Derivation of the supercurrent density

In this section, we will derive how to calculate the charge supercurrent associated with the finite center-of-mass momentum of Cooper pairs under the FMP constraint. We start with the definition of the current operator $\hat{\boldsymbol{j}}$. Generally, a current $\boldsymbol{j}$ in a system is induced by the change of the local polarization $\boldsymbol{P}$. The polarization operator is given by

$$\hat{\boldsymbol{P}} = e \sum_i \boldsymbol{R}_i c_i^\dagger c_i = e \sum_i \boldsymbol{R}_i n_i \tag{17}$$

for electrons of charge e sitting at a lattice site i (we suppress orbital and spin indices for now). The current is given by the time derivative (von-Neumann equation) of the polarization operator

$$\hat{\boldsymbol{j}} = \dot{\hat{\boldsymbol{P}}} = \frac{i}{\hbar}[\hat{\boldsymbol{P}}, H] \tag{18}$$

We want to study the Hamiltonian with a superconducting pairing field Δ_{ij}, where we here recast Eq. (16)

$$H = \underbrace{\sum_{ij} t_{ij} c_i^\dagger c_j}_{H_{\mathrm{N}} \equiv H_0} + \underbrace{\sum_{ij} \Delta_{ij} c_i c_j + \Delta_{ij}^* c_j^\dagger c_i^\dagger}_{H_{\mathrm{AN}} \equiv H_{\mathrm{SC}}} \tag{19}$$

for discrete lattice sites i instead of the continuous positions $\boldsymbol{r}$. To evaluate expression (18), we have to solve three kinds of commutators

$$\begin{aligned}
[n_m, c_i^\dagger c_j] &= c_i^\dagger [n_m, c_j] + [n_m, c_i^\dagger] c_j = (\delta_{mi} - \delta_{mj}) c_i^\dagger c_j \\
[n_m, c_i c_j] &= c_i [n_m, c_j] + [n_m, c_i] c_j = -(\delta_{mi} + \delta_{mj}) c_i c_j \\
[n_m, c_i^\dagger c_j^\dagger] &= c_i^\dagger [n_m, c_j^\dagger] + [n_m, c_i^\dagger] c_j^\dagger = (\delta_{mi} + \delta_{mj}) c_i^\dagger c_j^\dagger
\end{aligned}$$

where we used $[A, BC] = B[A, C] + [A, B]C$ and $[n_m, c_i^\dagger] = \delta_{im} c_i^\dagger$ ($[n_m, c_i] = -\delta_{im} c_i$). We inspect the normal and anomalous component separately ($\hat{\boldsymbol{j}} = \hat{\boldsymbol{j}}_{\mathrm{N}} + \hat{\boldsymbol{j}}_{\mathrm{AN}}$):

$$\begin{aligned}
\hat{\boldsymbol{j}}_{\mathrm{N}} = \frac{i}{\hbar}[\hat{\boldsymbol{P}}, H_{\mathrm{N}}] &= i\frac{e}{\hbar} \sum_{ijm} \boldsymbol{R}_m t_{ij} [n_m, c_i^\dagger c_j] = i\frac{e}{\hbar} \sum_{ijm} \boldsymbol{R}_m t_{ij} (\delta_{mi} - \delta_{mj}) c_i^\dagger c_j \\
&= i\frac{e}{\hbar} \sum_{ij} (\boldsymbol{R}_i - \boldsymbol{R}_j) t_{ij} c_i^\dagger c_j
\end{aligned} \tag{20}$$

$$\begin{aligned}
\hat{\boldsymbol{j}}_{\mathrm{AN}} = \frac{i}{\hbar}[\hat{\boldsymbol{P}}, H_{\mathrm{AN}}] &= i\frac{e}{\hbar} \sum_{ijm} \boldsymbol{R}_m (\Delta_{ij} [n_m, c_i c_j] + \Delta_{ij}^* [n_m, c_j^\dagger c_i^\dagger]) \\
&= -i\frac{e}{\hbar} \sum_{ijm} \boldsymbol{R}_m (\delta_{mi} + \delta_{mj}) (\Delta_{ij} c_i c_j - \Delta_{ij}^* c_j^\dagger c_i^\dagger) \\
&= -i\frac{e}{\hbar} \sum_{ij} (\boldsymbol{R}_i + \boldsymbol{R}_j) (\Delta_{ij} c_i c_j - \Delta_{ij}^* c_j^\dagger c_i^\dagger)
\end{aligned} \tag{21}$$

For calculating the current density $\boldsymbol{j} = \langle \hat{\boldsymbol{j}} \rangle$ we can make simplifications using the fact that we have local s-wave pairing in our system, i.e., $\Delta_{ij} \equiv \delta_{ij}\Delta e^{i\boldsymbol{q}\boldsymbol{R}_i}$. Since $\langle c_i c_j \rangle = -\langle c_j c_i \rangle$ and $\Delta_{ij} = \Delta_{ji}$, the expectation value of the anomalous part $\langle \hat{\boldsymbol{j}}_{\mathrm{AN}} \rangle$ vanishes then.

Since the anomalous part does not contribute, we only have to evaluate the normal component (20) of the current. For this purpose, we we will assume that the states at site i represent Wannier orbitals $i \rightarrow (\boldsymbol{R}_i, \alpha_i, \sigma_i)$ (orbital α, spin σ) which are centered on the unit cell center as is the case in the A_3C_{60} model (c.f. Note 4-D). Then, we can insert the Fourier transform of the creation and annihilation operators

$$c_i = \frac{1}{N_{\boldsymbol{k}}} \sum_{\boldsymbol{k}} \langle i|\boldsymbol{k}\rangle c_{\boldsymbol{k}} = \sum_{\boldsymbol{k}} e^{-i\boldsymbol{k}\boldsymbol{R}_i} \delta_{\alpha_i,\alpha_{\boldsymbol{k}}} \delta_{\sigma_i,\sigma_{\boldsymbol{k}}} c_{\boldsymbol{k}} \,, \qquad c_i^\dagger = \frac{1}{N_{\boldsymbol{k}}} \sum_{\boldsymbol{k}} e^{i\boldsymbol{k}\boldsymbol{R}_i} \delta_{\alpha_i,\alpha_{\boldsymbol{k}}} \delta_{\sigma_i,\sigma_{\boldsymbol{k}}} c_{\boldsymbol{k}}^\dagger \tag{22}$$

to yield

$$\begin{aligned}
\hat{\boldsymbol{j}} = \hat{\boldsymbol{j}}_{\mathrm{N}} =& i\frac{e}{\hbar}\frac{1}{N_{\boldsymbol{k}}^2} \sum_{ij\boldsymbol{k}\boldsymbol{k}'} \delta_{\sigma_i,\sigma}\delta_{\sigma_j,\sigma'}\delta_{\alpha_i,\alpha}\delta_{\alpha_j,\alpha'}\delta_{\sigma_i\sigma_j} [\boldsymbol{R}_i - \boldsymbol{R}_j] t_{\alpha_i\alpha_j}(\boldsymbol{R}_i - \boldsymbol{R}_j) e^{i(\boldsymbol{k}\boldsymbol{R}_i - \boldsymbol{k}'\boldsymbol{R}_j)} c_{\boldsymbol{k}\alpha\sigma}^\dagger c_{\boldsymbol{k}'\alpha'\sigma'} \\
\overset{\boldsymbol{R}_i \mapsto \boldsymbol{R}_i + \boldsymbol{R}_j}{=}& i\frac{e}{\hbar N_{\boldsymbol{k}}} \sum_{\substack{\boldsymbol{R}_i\boldsymbol{k}\boldsymbol{k}' \\ \alpha\alpha'\sigma}} \boldsymbol{R}_i t_{\alpha\alpha'}(\boldsymbol{R}_i) e^{i\boldsymbol{k}\boldsymbol{R}_i} \underbrace{\frac{1}{N_{\boldsymbol{k}}} \sum_{\boldsymbol{R}_j} e^{i(\boldsymbol{k}-\boldsymbol{k}')\boldsymbol{R}_j}}_{\delta_{\boldsymbol{k}\boldsymbol{k}'}} c_{\boldsymbol{k}\alpha\sigma}^\dagger c_{\boldsymbol{k}'\alpha'\sigma} \\
=& \frac{e}{\hbar N_{\boldsymbol{k}}} \sum_{\boldsymbol{k}\alpha\alpha'\sigma} \underbrace{i \sum_{\boldsymbol{R}_i} \boldsymbol{R}_i t_{\alpha\alpha'}(\boldsymbol{R}_i) e^{i\boldsymbol{k}\boldsymbol{R}_i}}_{=(\nabla_{\boldsymbol{k}} h(\boldsymbol{k}))_{\alpha\alpha'}} c_{\boldsymbol{k}\alpha\sigma}^\dagger c_{\boldsymbol{k}\alpha'\sigma} = \frac{e}{N_{\boldsymbol{k}}} \sum_{\boldsymbol{k}\alpha\alpha'\sigma} \boldsymbol{v}_{\alpha\alpha'}(\boldsymbol{k}) c_{\boldsymbol{k}\alpha\sigma}^\dagger c_{\boldsymbol{k}\alpha'\sigma}
\end{aligned} \tag{23}$$

with the velocity $\boldsymbol{v}(\boldsymbol{k}) = \frac{1}{\hbar}\nabla_{\boldsymbol{k}} h(\boldsymbol{k})$. Thus, the current density is given by

$$\boldsymbol{j}_{\boldsymbol{q}} = \langle \hat{\boldsymbol{j}} \rangle_{\boldsymbol{q}} = \frac{e}{N_{\boldsymbol{k}}} \sum_{\boldsymbol{k}\alpha\gamma\sigma} \boldsymbol{v}_{\alpha\gamma}(\boldsymbol{k}) \langle c_{\boldsymbol{k}\alpha\sigma}^\dagger c_{\boldsymbol{k}\gamma\sigma} \rangle_{\boldsymbol{q}} = \frac{2e}{N_{\boldsymbol{k}}} \sum_{\boldsymbol{k}\alpha\gamma} \boldsymbol{v}_{\alpha\gamma}(\boldsymbol{k}) \langle c_{\boldsymbol{k}\alpha\uparrow}^\dagger c_{\boldsymbol{k}\gamma\uparrow} \rangle_{\boldsymbol{q}} \tag{24}$$

where we introduced the index $\boldsymbol{q}$ to the expectation value $\langle \ldots \rangle_{\boldsymbol{q}}$ to stress that the reduced density matrix $\langle c_{\boldsymbol{k}\alpha\uparrow}^\dagger c_{\boldsymbol{k}\gamma\uparrow} \rangle_{\boldsymbol{q}}$ is evaluated for the FMP constraint imposed on the gap and order parameter, i.e., $\Delta e^{i\boldsymbol{q}\boldsymbol{R}_n}$. We connect to Green function theories by writing

$$\langle c_{\boldsymbol{k}\alpha\uparrow}^\dagger c_{\boldsymbol{k}\gamma\uparrow} \rangle_{\boldsymbol{q}} = \langle c_{\boldsymbol{k}-\frac{\boldsymbol{q}}{2}+\frac{\boldsymbol{q}}{2}\alpha\uparrow}^\dagger c_{\boldsymbol{k}-\frac{\boldsymbol{q}}{2}+\frac{\boldsymbol{q}}{2}\gamma\uparrow} \rangle_{\boldsymbol{q}} = \left[\underline{G}_{\boldsymbol{q}} \left(\tau = 0^-, \boldsymbol{k} - \frac{\boldsymbol{q}}{2} \right) \right]_{\gamma\alpha} = \left[\underline{\mathcal{G}}_{\boldsymbol{q}}^{\uparrow\uparrow} \left(\tau = 0^-, \boldsymbol{k} - \frac{\boldsymbol{q}}{2} \right) \right]_{\gamma\alpha} \tag{25}$$

We want to stress that the velocity $\boldsymbol{v}(\boldsymbol{K})$ and the reduced density matrix $\langle c_{\boldsymbol{K}\sigma}^\dagger c_{\boldsymbol{K}\sigma} \rangle$ have to carry the same momentum label $\boldsymbol{K}$ to fulfill Eq. (24) (here $\boldsymbol{K} = \boldsymbol{k}$). The expression in terms of a Green function then depends decisively on the notation used for FMP in the Nambu–Gor'kov

formalism. To match the definition given in Eq. (34) (and Eq. (4) of the main text) necessitates a shift of the $\boldsymbol{k}$ argument.

Thus, we obtain an expression for the current density derived from the Nambu–Gor'kov Green function as stated in Eq. (6) of the main text:

$$\boldsymbol{j}_{\boldsymbol{q}} = \frac{2e}{N_{\boldsymbol{k}}} \sum_{\boldsymbol{k}\alpha\gamma} \boldsymbol{v}_{\alpha\gamma}(\boldsymbol{k}) \left[\underline{G}_{\boldsymbol{q}} \left(\tau = 0^{-}, \boldsymbol{k} - \frac{\boldsymbol{q}}{2} \right) \right]_{\gamma\alpha} = \frac{2e}{N_{\boldsymbol{k}}} \sum_{\boldsymbol{k}} \mathrm{Tr}_{\alpha} \left[\underline{\boldsymbol{v}}(\boldsymbol{k}) \underline{G}_{\boldsymbol{q}} \left(\tau = 0^{-}, \boldsymbol{k} - \frac{\boldsymbol{q}}{2} \right) \right] \tag{26}$$

In practical calculations, however, we use Eq. (40) as this shows better convergence with respect to the Matsubara summation associated with obtaining $G(\tau = 0^{-})$. For details, see Note 4-C. We note that our approach reduces to linear-response-based approaches to calculate the stiffness $D_{\mathrm{s}} \propto \lambda_{\mathrm{L}}^{-2}$ in the limit of $q \to 0$. In this limit, one finds $\boldsymbol{j} = -D_{\mathrm{s}}\boldsymbol{A}$ by introducing a small $\boldsymbol{A}$ via Peierls-substitution [47] which is gauge-equivalent to the FMP constraint with small $\boldsymbol{q}$ [62]. We, however, explicitly account for finite q in our calculations inside the superconducting phase and determine the depairing current j_{dp} for the evaluation of λ_{L} (see Note 5-B). We stress that one also needs the full q-dependence encoded in the order parameter to evaluate the correlation length $\xi(T)$.

We want to note that a similar expression to Eq. (26) is given above Eq. (38.13) in the book by Abrikosov, Gor'kov, and Dzyaloshinski [60] as well as in Eq. (14.245) in the book by Coleman [4]. In both cases, however, it is discussed in the context of an external magnetic field $\boldsymbol{A}$ similar to the implementation in Ref. [45].

Supplementary Note 4: Numerical implementation of DMFT with FMP constraint

4-A Nambu–Gor'kov formalism with finite-momentum pairing

We want to comment on the Dynamical Mean-Field theory (DMFT) calculations in Nambu space under the constraint of finite-momentum pairing (FMP). First, we summarize the description of superconductivity within DMFT using the Nambu–Gor'kov formalism entering explicitly the superconducting state for zero-momentum pairing [51]. Afterwards, we detail how the FMP constraint can be incorporated into the superconducting Nambu Gor'kov DMFT formalism.

To extend the normal state DMFT formalism to Nambu–Gor'kov space [51], we perform a particle-hole transformation of the spin-down sector $c^{\dagger}_{\boldsymbol{k}\alpha\downarrow} \mapsto c_{-\boldsymbol{k}\alpha\downarrow}$ and introduce Nambu spinors

$$\psi^{\dagger}_{\boldsymbol{k},\alpha} = \begin{pmatrix} c^{\dagger}_{\boldsymbol{k}\alpha\uparrow} & c_{-\boldsymbol{k}\alpha\downarrow} \end{pmatrix}, \quad \psi_{\boldsymbol{k},\alpha} = \begin{pmatrix} c_{\boldsymbol{k}\alpha\uparrow} \\ c^{\dagger}_{-\boldsymbol{k}\alpha\downarrow} \end{pmatrix} \tag{27}$$

The corresponding single-particle Green function (Nambu–Gor'kov Green function) becomes a 2×2 matrix in Nambu space

$$\begin{aligned} \mathcal{G}_{\alpha\gamma}(\tau, \boldsymbol{k}) = -\langle T_\tau \psi_{\boldsymbol{k},\alpha}(\tau)\psi^\dagger_{\boldsymbol{k},\gamma}\rangle &= \begin{pmatrix} -\langle T_\tau c_{\boldsymbol{k}\alpha\uparrow}(\tau) c^\dagger_{\boldsymbol{k}\gamma\uparrow}\rangle & -\langle T_\tau c_{\boldsymbol{k}\alpha\uparrow}(\tau) c_{-\boldsymbol{k}\gamma\downarrow}\rangle \\ -\langle T_\tau c^\dagger_{-\boldsymbol{k}\alpha\downarrow}(\tau) c^\dagger_{\boldsymbol{k}\gamma\uparrow}\rangle & -\langle T_\tau c^\dagger_{-\boldsymbol{k}\alpha\downarrow}(\tau) c_{-\boldsymbol{k}\gamma\downarrow}\rangle \end{pmatrix} \\ &= \begin{pmatrix} G_{\alpha\gamma}(\tau,\boldsymbol{k}) & F_{\alpha\gamma}(\tau,\boldsymbol{k}) \\ F^\dagger_{\alpha\gamma}(\tau,\boldsymbol{k}) & \bar{G}_{\alpha\gamma}(\tau,-\boldsymbol{k}) \end{pmatrix} = \begin{pmatrix} G_{\alpha\gamma}(\tau,\boldsymbol{k}) & F_{\alpha\gamma}(\tau,\boldsymbol{k}) \\ F^\dagger_{\alpha\gamma}(\tau,\boldsymbol{k}) & -G_{\alpha\gamma}(-\tau,-\boldsymbol{k}) \end{pmatrix} \equiv \begin{pmatrix} \mathcal{G}^{\uparrow\uparrow}_{\alpha\gamma} & \mathcal{G}^{\uparrow\downarrow}_{\alpha\gamma} \\ \mathcal{G}^{\downarrow\uparrow}_{\alpha\gamma} & \mathcal{G}^{\downarrow\downarrow}_{\alpha\gamma} \end{pmatrix} \end{aligned} \quad (28)$$

where we used $\underline{\bar{G}}(\tau, -\boldsymbol{k}) = -\underline{G}(-\tau, -\boldsymbol{k})$ for the hole propagator and F denotes the anomalous (Gor'kov) Green function. The Nambu–Gor'kov Green function is determined from a Dyson equation where the non-interacting Green function

$$[\underline{\mathcal{G}}^0(i\omega_n, \boldsymbol{k})]^{-1} = \begin{pmatrix} (i\omega_n + \mu)\mathbb{1} - \underline{h}(\boldsymbol{k}) & 0 \\ 0 & (i\omega_n - \mu)\mathbb{1} + \underline{h}(-\boldsymbol{k}) \end{pmatrix} \equiv i\omega_n \mathbb{1} \cdot \sigma_0 - [\underline{h}(\boldsymbol{k}) - \mu\mathbb{1}] \cdot \sigma_z \quad (29)$$

and self-energy

$$\underline{\mathcal{S}}(i\omega_n) = \begin{pmatrix} \underline{\Sigma}^{\mathrm{N}}(i\omega_n) & \underline{\Sigma}^{\mathrm{AN}}(i\omega_n) \\ \underline{\Sigma}^{\mathrm{AN}}(i\omega_n) & -[\underline{\Sigma}^{\mathrm{N}}]^*(i\omega_n) \end{pmatrix} \equiv \Re\underline{\Sigma}^{\mathrm{N}}(i\omega_n) \cdot \sigma_z + i\Im\underline{\Sigma}^{\mathrm{N}}(i\omega_n) \cdot \sigma_0 + \underline{\Sigma}^{\mathrm{AN}}(i\omega_n) \cdot \sigma_x \quad (30)$$

also become matrices in Nambu space which can be expressed by Pauli matrices σ_i ($i = 0, x, y, z$) for inversion symmetry $h(\boldsymbol{k}) = h(-\boldsymbol{k})$. The self-energy $\mathcal{S}$ is obtained from solving the appropriate impurity problem defined by the Weiss field $\mathcal{G}_{\mathrm{W}}$ in Nambu space and the particle-hole-transformed interaction Hamiltonion. In addition to the normal component $\Sigma^{\mathrm{N}} \equiv \Sigma$, the self-energy gains an anomalous matrix element Σ^{AN} for which the gauge is chosen such that it is real-valued, i.e., only σ_x is involved in constructing $\mathcal{S}$. Thus, the lattice Green function in Nambu–Gor'kov space is given by

$$\begin{aligned} [\underline{\mathcal{G}}(i\omega_n, \boldsymbol{k})]^{-1} &= [\underline{\mathcal{G}}^0(i\omega_n, \boldsymbol{k})]^{-1} - \underline{\mathcal{S}}(i\omega_n) \\ &= \begin{pmatrix} (i\omega_n + \mu)\mathbb{1} - \underline{h}(\boldsymbol{k}) - \underline{\Sigma}^{\mathrm{N}}(i\omega_n) & -\underline{\Sigma}^{\mathrm{AN}}(i\omega_n) \\ -\underline{\Sigma}^{\mathrm{AN}}(i\omega_n) & (i\omega_n - \mu)\mathbb{1} + \underline{h}(-\boldsymbol{k}) + [\underline{\Sigma}^{\mathrm{N}}]^*(i\omega_n) \end{pmatrix} \end{aligned} \quad (31)$$

The self-consistency circle of DMFT (Eq. (9) in the Methods section) generally becomes a matrix formulation in Nambu space where the lattice Green function is replaced by the Nambu–Gor'kov Green function $\underline{G}_{\boldsymbol{k}}(i\omega_n) \mapsto \underline{\mathcal{G}}(i\omega_n, \boldsymbol{k})$. We can restate the DMFT self-consistency problem in the superconducting state using calligraphic letters

$$\begin{cases} \underline{\mathcal{G}}_{\mathrm{loc}}(i\omega_n) = \dfrac{1}{N_{\boldsymbol{k}}} \displaystyle\sum_{\boldsymbol{k}} \underline{\mathcal{G}}(i\omega_n, \boldsymbol{k}) \\ \underline{\mathcal{G}}^{-1}_{\mathrm{W}}(i\omega_n) = \underline{\mathcal{G}}^{-1}_{\mathrm{loc}}(i\omega_n) + \underline{\mathcal{S}}(i\omega_n) \\ \underline{\mathcal{S}}(i\omega_n) = \underline{\mathcal{G}}^{-1}_{\mathrm{W}}(i\omega_n) - \underline{\mathcal{G}}^{-1}_{\mathrm{imp}}(\omega_n) \end{cases} \quad (32)$$

We now want to incorporate the FMP constraint into the Nambu–Gor'kov formalism. To treat the phase $\mathrm{e}^{i\boldsymbol{q}\boldsymbol{r}}$ of the OP and gap in the framework of DMFT, several possibilities exist. For the simplest implementation, we introduce a phase gauge shift that cancels the momentum dependence of the order parameter $\Psi_{\boldsymbol{q}}(\boldsymbol{R}_i) = |\Psi|\mathrm{e}^{i\boldsymbol{q}\boldsymbol{R}_i} = \langle c_{i\uparrow}c_{i\downarrow}\rangle$ at site $\boldsymbol{R}_i$. By applying the transformation $c_{i\alpha\sigma} \mapsto c_{i\alpha\sigma}\mathrm{e}^{i\boldsymbol{q}\boldsymbol{R}_i/2}$ ($c^\dagger_{i,\sigma} \mapsto c^\dagger_{i,\sigma}\mathrm{e}^{-i\boldsymbol{q}\boldsymbol{R}_i/2}$), the hopping amplitudes $t(\boldsymbol{R}_{ij})$ are modified to yield $\tilde{t}(\boldsymbol{R}_{ij}) = t(\boldsymbol{R}_{ij})\mathrm{e}^{i\boldsymbol{q}\boldsymbol{R}_{ij}/2}$ such that the dispersion $\underline{\varepsilon}_{\boldsymbol{k}}$ obtained from diagonalizing $\underline{h}(\boldsymbol{k})$ is effectively replaced by $\underline{\varepsilon}_{\boldsymbol{k}\pm\boldsymbol{q}/2}$, i.e., the dispersion for up and down spins gets shifted by $\pm\boldsymbol{q}/2$, respectively. This shows that the introduction of FMP breaks time-reversal symmetry. The advantage of completely transferring the $\boldsymbol{q}$-dependence to the hopping matrix is that we keep the gauge freedom to choose the anomalous self-energy to be a real-valued function.

To this end, we recast Eqs. (27) to (31) including the FMP momentum $\boldsymbol{q}$. Through the gauge transform, the Fourier transformed creation and annihilation operators pick up the $\pm\boldsymbol{q}/2$ momentum shift such that the Nambu spinors obtain an additional parametric dependence

$$\psi^\dagger_{\boldsymbol{k},\boldsymbol{q},\alpha} = \begin{pmatrix} c^\dagger_{\boldsymbol{k}+\frac{\boldsymbol{q}}{2}\alpha\uparrow} & c_{-\boldsymbol{k}+\frac{\boldsymbol{q}}{2}\alpha\downarrow} \end{pmatrix} \quad , \quad \psi_{\boldsymbol{k}+\frac{\boldsymbol{q}}{2},\alpha} = \begin{pmatrix} c_{\boldsymbol{k}+\frac{\boldsymbol{q}}{2}\alpha\uparrow} \\ c^\dagger_{-\boldsymbol{k}+\frac{\boldsymbol{q}}{2}\alpha\downarrow} \end{pmatrix} \tag{33}$$

The Nambu–Gor'kov Green function consequently is parameterized by $\boldsymbol{q}$ as well

$$\begin{aligned}\left[\underline{\mathcal{G}}_{\boldsymbol{q}}(\tau,\boldsymbol{k})\right]_{\alpha\gamma} &= -\langle T_\tau \psi_{\boldsymbol{k},\boldsymbol{q},\alpha}(\tau)\psi^\dagger_{\boldsymbol{k},\boldsymbol{q},\gamma}\rangle = \begin{pmatrix} -\langle T_\tau c_{\boldsymbol{k}+\frac{\boldsymbol{q}}{2}\alpha\uparrow}(\tau)c^\dagger_{\boldsymbol{k}+\frac{\boldsymbol{q}}{2}\gamma\uparrow}\rangle & -\langle T_\tau c_{\boldsymbol{k}+\frac{\boldsymbol{q}}{2}\alpha\uparrow}(\tau)c_{-\boldsymbol{k}+\frac{\boldsymbol{q}}{2}\gamma\downarrow}\rangle \\ -\langle T_\tau c^\dagger_{-\boldsymbol{k}+\frac{\boldsymbol{q}}{2}\alpha\downarrow}(\tau)c^\dagger_{\boldsymbol{k}+\frac{\boldsymbol{q}}{2}\gamma\uparrow}\rangle & -\langle T_\tau c^\dagger_{-\boldsymbol{k}+\frac{\boldsymbol{q}}{2}\alpha\downarrow}(\tau)c_{-\boldsymbol{k}+\frac{\boldsymbol{q}}{2}\gamma\downarrow}\rangle \end{pmatrix} \\ &= \begin{pmatrix} \left[\underline{G}_{\boldsymbol{q}}(\tau,\boldsymbol{k})\right]_{\alpha\gamma} & \left[\underline{F}_{\boldsymbol{q}}(\tau,\boldsymbol{k})\right]_{\alpha\gamma} \\ \left[\underline{F}^\dagger_{\boldsymbol{q}}(\tau,\boldsymbol{k})\right]_{\alpha\gamma} & \left[\underline{\bar{G}}_{\boldsymbol{q}}(\tau,-\boldsymbol{k})\right]_{\alpha\gamma} \end{pmatrix} \equiv \begin{pmatrix} \left[\underline{\mathcal{G}}^{\uparrow\uparrow}_{\boldsymbol{q}}(\tau,\boldsymbol{k})\right]_{\alpha\gamma} & \left[\underline{\mathcal{G}}^{\uparrow\downarrow}_{\boldsymbol{q}}(\tau,\boldsymbol{k})\right]_{\alpha\gamma} \\ \left[\underline{\mathcal{G}}^{\downarrow\uparrow}_{\boldsymbol{q}}(\tau,\boldsymbol{k})\right]_{\alpha\gamma} & \left[\underline{\mathcal{G}}^{\downarrow\downarrow}_{\boldsymbol{q}}(\tau,\boldsymbol{k})\right]_{\alpha\gamma} \end{pmatrix}\end{aligned} \tag{34}$$

Generally, it holds that $\underline{\mathcal{G}}^{\uparrow\uparrow}_{\boldsymbol{q}}(\tau,\boldsymbol{k}) \neq -\underline{\mathcal{G}}^{\downarrow\downarrow}_{\boldsymbol{q}}(-\tau,-\boldsymbol{k})$ for arbitrary, finite $\boldsymbol{q}$ due to the time-reversal symmetry breaking. Note that it is possible to define the Fourier transform of $c_i^{(\dagger)}$ differently such that the pairing is non-symmetric with respect to $\boldsymbol{q}$. Another often employed notation describes Cooper pairs with electrons of momenta $\boldsymbol{k}$ (in $\mathcal{G}^{\uparrow\uparrow}$) and $-\boldsymbol{k}+\boldsymbol{q}$ (in $\mathcal{G}^{\downarrow\downarrow}$) (as depicted in Fig. 2 of the main text). We here choose the symmetric notation by putting $-\frac{\boldsymbol{q}}{2}$ to both diagonal components.

On Matsubara frequencies, the Nambu–Gor'kov Green function is set up via (c.f. Eq. (10) in the main text)

$$[\underline{\mathcal{G}}_{\boldsymbol{q}}(i\omega_n,\boldsymbol{k})]^{-1} = \begin{pmatrix} (i\omega_n+\mu)\mathbb{1} - \underline{h}(\boldsymbol{k}+\frac{\boldsymbol{q}}{2}) - \underline{\Sigma}^{\mathrm{N}}(i\omega_n) & -\underline{\Sigma}^{\mathrm{AN}}(i\omega_n) \\ -\underline{\Sigma}^{\mathrm{AN}}(i\omega_n) & (i\omega_n-\mu)\mathbb{1} + \underline{h}(-\boldsymbol{k}+\frac{\boldsymbol{q}}{2}) + [\underline{\Sigma}^{\mathrm{N}}]^*(i\omega_n) \end{pmatrix} \tag{35}$$

We note that the approach outlined here does not require the computationally more demanding use of supercells as, e.g., implemented in Refs. [101, 102] and discussed in the review by Kinnunen et al. [59] to study FFLO-type superconductivity.

To induce symmetry-breaking in our calculations, we add a small pairing field $\eta = 0.1$ meV on the off-diagonal of the Nambu–Gor'kov Green function throughout the calculation. We keep it for the whole superconducting DMFT loop because it helps stabilizing the calculations. We checked that the presence of the small, but finite η does not change the results. In this work, we always choose $\boldsymbol{q} = q\boldsymbol{b}_1$ along the direction of a reciprocal lattice vector $\boldsymbol{b}_1$ (c.f. Note 4-D). The calculations with finite q are performed in practice as in the case of $q = 0$, but with q becoming an additional input parameter. Calculations can be sped up by first converging the DMFT loop for $q = 0$ and then using the result as a starting point for finite $q > 0$ values.

4-B Determination of chemical potential

In our calculations, we adjust the chemical potential μ in each DMFT iteration in order to keep the filling of the system fixed to $\langle n\rangle_{\boldsymbol{q}} = N_{\mathrm{orb}} = 3$, i.e., the t_{1u} bands of A_3C_{60} are half-filled. The code implementation was done in Ref. [67] (see also Ref. [68]) for DMFT in the Nambu–Gor'kov formalism with $q = 0$, but it can also be used for finite momenta. To determine the chemical potential, we solve the following equation

$$\begin{aligned}\langle n\rangle_{\boldsymbol{q}} &= \frac{1}{N_{\boldsymbol{k}}}\sum_{\boldsymbol{k}\alpha\sigma}\langle c^{\dagger}_{\boldsymbol{k}+\frac{\boldsymbol{q}}{2}\alpha\sigma}c_{\boldsymbol{k}+\frac{\boldsymbol{q}}{2}\alpha\sigma}\rangle = \frac{1}{N_{\boldsymbol{k}}}\sum_{\boldsymbol{k}\alpha}\langle c^{\dagger}_{\boldsymbol{k}+\frac{\boldsymbol{q}}{2}\alpha\uparrow}c_{\boldsymbol{k}+\frac{\boldsymbol{q}}{2}\alpha\uparrow}\rangle + \langle c^{\dagger}_{-\boldsymbol{k}+\frac{\boldsymbol{q}}{2}\alpha\downarrow}c_{-\boldsymbol{k}+\frac{\boldsymbol{q}}{2}\alpha\downarrow}\rangle \\ &= \frac{1}{N_{\boldsymbol{k}}}\sum_{\boldsymbol{k}\alpha}\langle 1 - c_{\boldsymbol{k}+\frac{\boldsymbol{q}}{2}\alpha\uparrow}c^{\dagger}_{\boldsymbol{k}+\frac{\boldsymbol{q}}{2}\alpha\uparrow}\rangle + \langle c^{\dagger}_{-\boldsymbol{k}+\frac{\boldsymbol{q}}{2}\alpha\downarrow}c_{-\boldsymbol{k}+\frac{\boldsymbol{q}}{2}\alpha\downarrow}\rangle \\ &= N_{\mathrm{orb}} + \frac{1}{N_{\boldsymbol{k}}}\sum_{\boldsymbol{k}\alpha}[\underline{G}_{\boldsymbol{q}}(\tau = 0^+, \boldsymbol{k}) - \underline{\bar{G}}_{\boldsymbol{q}}(\tau = 0^+, -\boldsymbol{k})]_{\alpha\alpha} \\ &= N_{\mathrm{orb}} + \frac{1}{N_{\boldsymbol{k}}}\sum_{\boldsymbol{k}\omega_n}\mathrm{Tr}_{\alpha}[\mathcal{G}^{\uparrow\uparrow}_{\boldsymbol{q}} - \mathcal{G}^{\downarrow\downarrow}_{\boldsymbol{q}}](i\omega_n, \boldsymbol{k})\mathrm{e}^{i\omega_n 0^+}\end{aligned} \tag{36}$$

In the second step, we relabeled momentum $\boldsymbol{k} \mapsto \boldsymbol{k} + \boldsymbol{q}$ for the spin down sector. Taking the difference $\mathcal{G}^{\uparrow\uparrow}_{\boldsymbol{q}} - \mathcal{G}^{\downarrow\downarrow}_{\boldsymbol{q}}$ helps with the convergence of the Matsubara sum to evaluate the Green functions at $\tau = 0^+$ since the frequency tail becomes $\mathcal{O}(1/(i\omega_n)^2)$.

4-C Handling the Matsubara summation in the calculation of the current density

The expression for the current density, Eq. (26), contains a Matsubara sum of the spin-up Nambu–Gor'kov Green function component $\mathcal{G}^{\uparrow\uparrow} = G$ to compute the reduced density matrix

$\langle c^\dagger_{\boldsymbol{k}\alpha\uparrow} c_{\boldsymbol{k}\gamma\uparrow}\rangle_{\boldsymbol{q}}$ (c.f. Eq. (25)). The Green function typically has slow convergence of Matsubara frequencies due to the $1/(i\omega_n)$-tail at large frequencies. We can achieve better convergence by including the inverse of the diagonal part of the Nambu Gor'kov Green function, i.e., the inverse of the non-interacting Green function plus the normal self-energy. Generally, we can expand the full Nambu Gor'kov Green function from Eq. (35) in the isospin space in terms of Pauli matrices

$$\underline{\mathcal{G}}^{-1} = \underline{g}_0\sigma_0 + \underline{g}_z\sigma_z + \underline{g}_x\sigma_x \tag{37}$$

We now define

$$\underline{\mathcal{G}}_{\mathrm{N}}^{-1} = \underline{g}_0\sigma_0 + \underline{g}_z\sigma_z \qquad \text{and} \qquad \underline{\mathcal{G}}_{\Delta}^{-1} = \underline{g}_x\sigma_x \tag{38}$$

Since $\mathcal{G}_{\mathrm{N}}$ describes a time-reversal symmetric system, the following term

$$\sum_{\boldsymbol{k}} \mathrm{Tr}_\alpha \left[\underline{\boldsymbol{v}}(\boldsymbol{k})\underline{\mathcal{G}}_{\mathrm{N}}^{\uparrow\uparrow}\left(\tau = 0^-, \boldsymbol{k} - \frac{\boldsymbol{q}}{2}\right)\right] = 0 \tag{39}$$

has to vanish. Eq. (39) essentially states that the charge supercurrent is induced by the superconducting condensate which only contributes to the full Nambu Green function $\mathcal{G}$ via the anomalous self-energy contained in $\mathcal{G}_{\Delta}^{-1}$ since $\underline{g}_x \equiv \underline{\Sigma}_{\mathrm{AN}}$. Thus, we can subtract Eq. (39) from the current to obtain

$$\boldsymbol{j} = \frac{2e}{N_{\boldsymbol{k}}}\sum_{\boldsymbol{k}} \mathrm{Tr}_\alpha \left[\underline{\boldsymbol{v}}(\boldsymbol{k})\left\{\underline{\mathcal{G}} - \underline{\mathcal{G}}_{\mathrm{N}}\right\}^{\uparrow\uparrow}\left(\tau = 0^-, \boldsymbol{k} - \frac{\boldsymbol{q}}{2}\right)\right] = \frac{2e}{N_{\boldsymbol{k}}}\sum_{\boldsymbol{k}} \mathrm{Tr}_\alpha \left[\underline{\boldsymbol{v}}(\boldsymbol{k})\delta\underline{\mathcal{G}}^{\uparrow\uparrow}\left(\tau = 0^-, \boldsymbol{k} - \frac{\boldsymbol{q}}{2}\right)\right] \tag{40}$$

which has a better convergence with respect to Matsubara frequencies, since $\delta\mathcal{G}^{\uparrow\uparrow} \propto 1/(i\omega_n)^3$ at large frequencies. To see the convergence behavior, we do a Taylor expansion where we focus on the isospin dependence only:

$$\begin{aligned}
\delta\mathcal{G} = \mathcal{G} - \mathcal{G}_{\mathrm{N}} &= (\mathcal{G}_{\mathrm{N}}^{-1} + G_{\Delta}^{-1})^{-1} - \mathcal{G}_{\mathrm{N}} = \mathcal{G}_{\mathrm{N}}(\sigma_0 + \mathcal{G}_{\Delta}^{-1}\mathcal{G}_{\mathrm{N}})^{-1} - \mathcal{G}_{\mathrm{N}} \\
&= \mathcal{G}_{\mathrm{N}}(\sigma_0 + \mathcal{G}_{\Delta}^{-1}\mathcal{G}_{\mathrm{N}} + \mathcal{G}_{\Delta}^{-1}\mathcal{G}_{\mathrm{N}}\mathcal{G}_{\Delta}^{-1}\mathcal{G}_{\mathrm{N}} + \ldots) - \mathcal{G}_{\mathrm{N}} \\
&= \mathcal{G}_{\mathrm{N}}\mathcal{G}_{\Delta}^{-1}\mathcal{G}_{\mathrm{N}} + \mathcal{G}_{\mathrm{N}}\mathcal{G}_{\Delta}^{-1}\mathcal{G}_{\mathrm{N}}\mathcal{G}_{\Delta}^{-1}\mathcal{G}_{\mathrm{N}} + \ldots
\end{aligned}$$

The first term of the last line does not have any diagonal components since $\mathcal{G}_{\mathrm{N}} = 1/\mathcal{G}_{\mathrm{N}}^{-1} \propto \ldots\sigma_0 + \ldots\sigma_z$ and $\mathcal{G}_{\Delta}^{-1} \propto \sigma_x$ such that their product

$$\mathcal{G}_{\mathrm{N}}\mathcal{G}_{\Delta}^{-1}\mathcal{G}_{\mathrm{N}} \propto \ldots\sigma_0\sigma_x + \ldots\sigma_x\sigma_z \propto \ldots\sigma_x + \ldots\sigma_y \tag{41}$$

has only off-diagonal components. Hence, the lowest order term contributing to the $\uparrow\uparrow$ component of $\delta\mathcal{G}$ is $O(\mathcal{G}_{\mathrm{N}}^3\mathcal{G}_{\Delta}^2)$ which has a $1/(i\omega_n)^3$-tail from $\mathcal{G}_{\mathrm{N}}^3$. The Taylor expansion shows

that $\mathcal{G}_\mathrm{N}$ is the zero-order term that causes the overall $1/(i\omega_n)$-tail of $\mathcal{G}$ which we mitigate with Eq. (40). An approximate expression for the current utilizing the Taylor expansion up to lowest order can be found in Eq. (38.13) in the book by Abrikosov, Gor'kov, and Dzyaloshinski [60].

We compute the momentum and Matsubara summation occurring in the expression of the supercurrent density $\boldsymbol{j}_{\boldsymbol{q}}$ (c.f. Eq. (26) in Note 4) using a 35^3 $\boldsymbol{k}$-mesh and 200 Matsubara frequencies.

4-D Lattice model details and current direction

We here give further details on the lattice model of A_3C_{60} materials derived in Ref. [79]. In this model, the C_{60} molecules reside on a fcc lattice for which we construct Bravais lattice vectors and momenta as

$$\boldsymbol{R}_i = \sum_{i=1}^{3} n_j \boldsymbol{a}_j \qquad , \qquad \boldsymbol{k} = \sum_{i=j}^{3} k_j \boldsymbol{b}_j \tag{42}$$

with $i \equiv (n_1, n_2, n_3)$. We choose the lattice and corresponding reciprocal lattice vectors to be

$$\boldsymbol{a}_1 = \frac{a}{2}(\hat{\boldsymbol{x}} + \hat{\boldsymbol{y}}) \;, \qquad \boldsymbol{a}_2 = \frac{a}{2}(\hat{\boldsymbol{y}} + \hat{\boldsymbol{z}}) \;, \qquad \boldsymbol{a}_3 = \frac{a}{2}(\hat{\boldsymbol{x}} + \hat{\boldsymbol{z}}) \tag{43}$$

$$\boldsymbol{b}_1 = \frac{2\pi}{a}(\hat{\boldsymbol{x}} + \hat{\boldsymbol{y}} - \hat{\boldsymbol{z}}) \;, \qquad \boldsymbol{b}_2 = \frac{2\pi}{a}(-\hat{\boldsymbol{x}} + \hat{\boldsymbol{y}} + \hat{\boldsymbol{z}}) \;, \qquad \boldsymbol{b}_3 = \frac{2\pi}{a}(\hat{\boldsymbol{x}} - \hat{\boldsymbol{y}} + \hat{\boldsymbol{z}}) \tag{44}$$

with lattice constant $a \sim 14.2 - 14.5$ Å. $\hat{\boldsymbol{x}}, \hat{\boldsymbol{y}}$, and $\hat{\boldsymbol{z}}$ are the Cartesian unit vectors. We restate the lattice model from Eq. (8) in the main text

$$H_\mathrm{kin} = \sum_{ij} \sum_{\alpha\gamma\sigma} t_{\alpha\gamma}(\boldsymbol{R}_{ij}) c^\dagger_{i\alpha\sigma} c_{j\gamma\sigma} = \sum_{\boldsymbol{k}} \sum_{\alpha\gamma\sigma} h_{\alpha\gamma}(\boldsymbol{k}) c^\dagger_{\boldsymbol{k}\alpha\sigma} c_{\boldsymbol{k}\gamma\sigma} \tag{45}$$

where we inserted the Fourier transformation (22) of creation and annihilation operators with $h_{\alpha\gamma}(\boldsymbol{k}) = \sum_j t_{\alpha\gamma}(\boldsymbol{R}_j) \mathrm{e}^{i\boldsymbol{k}\boldsymbol{R}_j}$. We here specify the hopping terms $t_{\alpha\gamma}(\boldsymbol{R}_{ij})$ connecting electrons of spin σ on sites i, j and molecular (Wannier) orbitals α, γ via $\boldsymbol{R}_{ij} = \boldsymbol{R}_i - \boldsymbol{R}_j$. The Wannier orbitals labeled $\alpha = 1, 2, 3$ describe degenerate p_x-, p_y-, and p_z-like orbitals. For the 12 nearest-neighbor (NN) distances, the hopping matrices are given by

$$\begin{pmatrix} t_1 & t_2 & 0 \\ t_2 & t_3 & 0 \\ 0 & 0 & t_4 \end{pmatrix} \text{ for } \boldsymbol{R} = (0.5,\ 0.5,\ 0.0), \qquad \begin{pmatrix} t_1 & -t_2 & 0 \\ -t_2 & t_3 & 0 \\ 0 & 0 & t_4 \end{pmatrix} \text{ for } \boldsymbol{R} = (0.5,\ -0.5,\ 0.0),$$

$$\begin{pmatrix} t_4 & 0 & 0 \\ 0 & t_1 & t_2 \\ 0 & t_2 & t_3 \end{pmatrix} \text{ for } \boldsymbol{R} = (0, 0,\ 0.5,\ 0.5), \qquad \begin{pmatrix} t_4 & 0 & 0 \\ 0 & t_1 & -t_2 \\ 0 & -t_2 & t_3 \end{pmatrix} \text{ for } \boldsymbol{R} = (0.0,\ 0.5, -0.5),$$

$$\begin{pmatrix} t_3 & 0 & t_2 \\ 0 & t_4 & 0 \\ t_2 & 0 & t_1 \end{pmatrix} \text{ for } \boldsymbol{R} = (0.5,\ 0.0,\ 0.5), \qquad \begin{pmatrix} t_3 & 0 & -t_2 \\ 0 & t_4 & 0 \\ -t_2 & 0 & t_1 \end{pmatrix} \text{ for } \boldsymbol{R} = (-0.5,\ 0.0,\ 0.5)$$

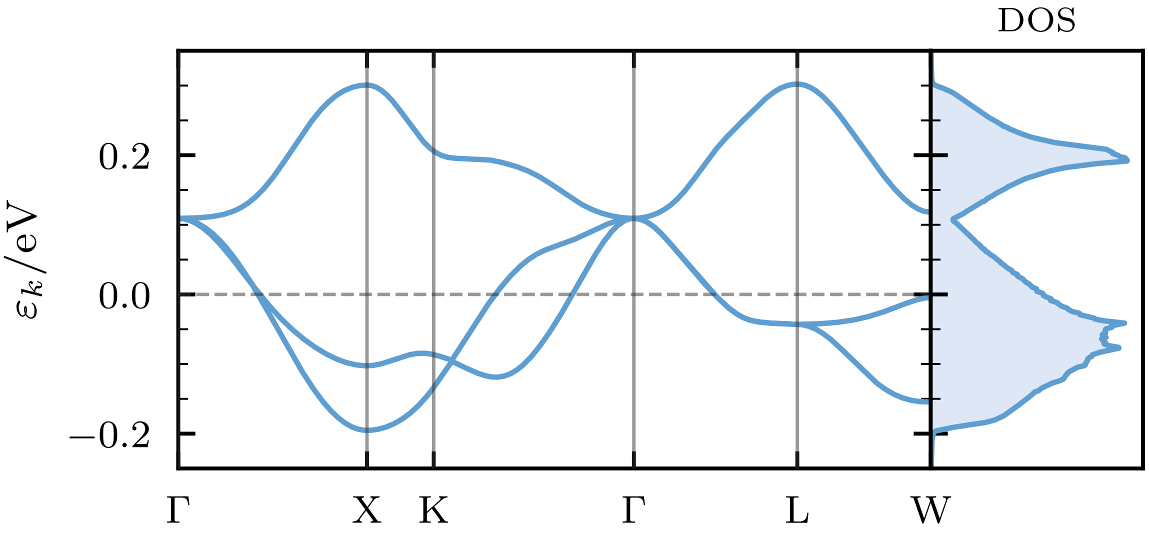

Supplementary Figure 2 | Electronic structure of fullerides. Band structure $\varepsilon_{\boldsymbol{k}}$ and density of states (DOS) of the degenerate half-filled t_{1u} bands using hopping parameters for K_3C_{60}.

where the connecting lattice vectors in Cartesian coordinates $\boldsymbol{R}_{ij} \equiv \boldsymbol{R} = (R_x,\ R_y,\ R_z)$ are in units of the lattice constant a. Hopping matrices for transfer processes to the 6 next-nearest-neighbor (NNN) sites of a C_{60} molecule are

$$\begin{pmatrix} t_5 & 0 & 0 \\ 0 & t_6 & 0 \\ 0 & 0 & t_7 \end{pmatrix} \text{ for } \boldsymbol{R} = (1,\ 0,\ 0),\quad \begin{pmatrix} t_7 & 0 & 0 \\ 0 & t_5 & 0 \\ 0 & 0 & t_6 \end{pmatrix} \text{ for } \boldsymbol{R} = (0,\ 1,\ 0),\quad \begin{pmatrix} t_6 & 0 & 0 \\ 0 & t_7 & 0 \\ 0 & 0 & t_5 \end{pmatrix} \text{ for } \boldsymbol{R} = (0,\ 0,\ 1)$$

The remaining NN and NNN hopping matrices can be generated from inversion symmetry $t_{\alpha\gamma}(\boldsymbol{R}) = t_{\alpha\gamma}(-\boldsymbol{R})$. In this work, we employ the Wannier construction for K_3C_{60} for which the numerical values are (in meV): $t_1 = -4$, $t_2 = -33.9$, $t_3 = 42.1$, $t_4 = -18.7$, $t_5 = -9.3$, $t_6 = -1.4$, $t_7 = -0.2$. The onsite energy $t_{\alpha\alpha}(\boldsymbol{R}_{ij} = \boldsymbol{0})$ is set to zero. We show the corresponding band structure (bandwidth $W \approx 0.5\,\text{eV}$) and density of states for the non-interacting model in Figure 2. The main difference between different A_3C_{60} compounds is the bandwidth W and effective electronic interaction strength U [68, 79]. One can approximate the volume effect induced by different alkali dopands by changing the ratio U/W. The interaction Hamiltonian H_{int} is discussed in more detail in Note 7.

We also comment on the direction of the current. In all calculations, we put the FMP momentum $\boldsymbol{q}$ parallel to one of the reciprocal lattice vectors: $\boldsymbol{q} = q\boldsymbol{b}_1 = \frac{2\pi q}{a}(\hat{\boldsymbol{x}} + \hat{\boldsymbol{y}} - \hat{\boldsymbol{z}})$ such that in Cartesian coordinates $q_x = q_y = -q_z$. By this, we can employ an analytical expression

for the velocity

$$\hbar \underline{v}(\boldsymbol{k}) = \nabla_{\boldsymbol{k}} \underline{h}(\boldsymbol{k}) = \nabla_{\boldsymbol{k}} \sum_{\boldsymbol{R}} \underline{t}(\boldsymbol{R}) \mathrm{e}^{i\boldsymbol{k}\boldsymbol{R}} = i \sum_{\boldsymbol{R}} \boldsymbol{R}\, \underline{t}(\boldsymbol{R}) \mathrm{e}^{i\boldsymbol{k}\boldsymbol{R}} \tag{46}$$

instead of numerically evaluating the gradient of $h(\boldsymbol{k})$ in Eq. (40). The direction of the velocity and, hence, the supercurrent can be used as an internal consistency check of the code. The condition of $\boldsymbol{j} \parallel \boldsymbol{q}$ demands that

$$\boldsymbol{j} = j_1 \boldsymbol{a}_1 + j_2 \boldsymbol{a}_2 + j_3 \boldsymbol{a}_3 \overset{!}{=} j(\boldsymbol{x} + \boldsymbol{y} - z) \tag{47}$$

$$= a(j_1 + j_3)\hat{\boldsymbol{x}} + a(j_1 + j_2)\hat{\boldsymbol{y}} + a(j_2 + j_3)\hat{\boldsymbol{z}} \tag{48}$$

$$\Leftrightarrow j_3 = j_2 = -\frac{1}{3} j_1 \tag{49}$$

To have the correct sign of the direction, we need $j_1 > 0$ (i.e., $j_2, j_3 < 0$). Since the fcc lattice has a very high symmetry, we can approximately treat the system to be isotropic. Because of this, we discuss in Fig. 2 of the main text and in Note 5-B only the absolute value of the current given by $|\boldsymbol{j}| = 2\sqrt{3}ax$ with $x = |j_2| = |j_3| = j_1/3$.

Supplementary Note 5: Details on the calculation of $|\Psi_q|$, ξ_0, j_{dp}, and λ_{L} from DMFT

In this section, we illustrate how the order parameter $|\Psi_q|$ and concomitantly the coherence length ξ_0 (c.f. Note 5-A) as well as the depairing current j_{dp} and London penetration depth λ_{L} (c.f. Note 5-B) are obtained from the $\boldsymbol{q}$- and T-dependence of the Nambu–Gor'kov Green function in practice. Furthermore, we elaborate on how the critical temperature extracted from the temperature dependence of $\xi(T)$ and $\lambda_{\mathrm{L}}(T)$ can be used to scrutinize the proximity region of the Mott insulating phase and how it impacts the superconducting region (c.f. Note 5-C and Fig. 4a of the main text).

5-A Order parameter and coherence length

Generally, the superconducting order parameter carries an orbital dependence. The superconducting pairing in A_3C_{60}, however, is orbital diagonal. Because of this, we perform an orbital average over the self-energy components Σ^{N} and Σ^{AN} in each iteration step of the DMFT loop such that they are diagonal matrices in orbital space with degenerate entries ($\Sigma^{(\mathrm{A})\mathrm{N}}_{\alpha\gamma} = \delta_{\alpha\gamma}\, \Sigma^{(\mathrm{A})\mathrm{N}}$). As a result, we explicitly prevent spontaneous orbital symmetry breaking in the self-energy [73] and the anomalous Green function F also becomes a degenerate, diagonal matrix in orbital space.

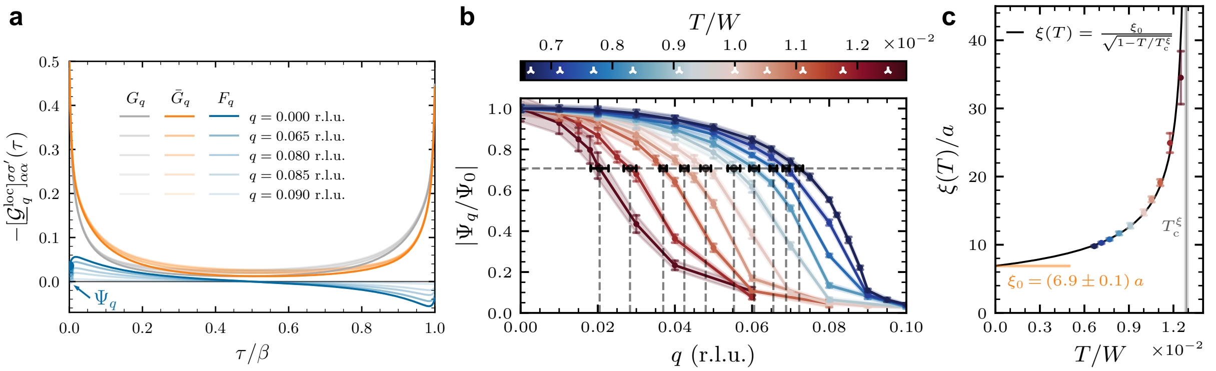

Supplementary Figure 3 | Order parameter $|\Psi_q|$ and coherence length ξ_0 from the local anomalous Green function F^{loc}. (**a**) Normal (particle $G_q = \mathcal{G}_q^{\uparrow\uparrow}$, hole $\bar{G}_q = \mathcal{G}_q^{\downarrow\downarrow}$) and anomalous ($F_q = \mathcal{G}_q^{\uparrow\downarrow}$) components of the local Nambu–Gor'kov Green function $\mathcal{G}_q^{\mathrm{loc}} = \sum_{\boldsymbol{k}} \mathcal{G}_q(\boldsymbol{k})$ for different values of $\boldsymbol{q}$ at fixed $T/W = 6.7\times10^{-3}$. The OP is taken at $\tau = 0$ which we indicate by an arrow. (**b**) Momentum dependence of the OP normalized to the $q = 0$ value for various T values. The condition $|\Psi_Q(T)/\Psi_0(T)| = 1/\sqrt{2}$ to determine the correlation length via $\xi(T) = 1/(\sqrt{2}Q)$ for fixed T is drawn with dashed lines. Different temperatures T are indicated in the color bar by white triangular markers and the shaded areas for each T show the range spanned by the uncertainty $\delta|\Psi_q|$ which we use for spline fitting to determine an error for $\xi(T)$. (**c**) Temperature dependence of $\xi(T)$ as obtained from panel **b** with the same coloring for each temperature. The fit of Eq. (3) (Eq. (2) in the main text) to extract ξ_0 is plotted with a solid black line. Shown data are results of the DMFT calculations for $U/W = 1.4$ and $J/W = -0.04$, equal to the content of Figs. 2 and 3 of the main text.

This allows us to work with a single-component OP for which we take the local anomalous Green function (c.f. Eq. (5) of the main text)

$$|\Psi_{\boldsymbol{q}}| \equiv [\underline{F}_{\boldsymbol{q}}^{\mathrm{loc}}(\tau = 0^-)]_{\alpha\alpha} = \sum_{\boldsymbol{k}} \langle c_{\alpha \boldsymbol{k}+\frac{\boldsymbol{q}}{2}\uparrow} c_{\alpha -\boldsymbol{k}+\frac{\boldsymbol{q}}{2}\downarrow} \rangle \tag{50}$$

Another option to define the OP is the superconducting gap Δ, c.f. Note 6. Since, here, F and Δ are orbital diagonal, they can be equivalently used for defining the OP as they have the same $\boldsymbol{q}$- and T-dependence. Taking Δ as the OP would change the relative scaling of the GL free energy because of $\Delta \approx \mathcal{U}_{\mathrm{eff}} F$ with an effective pairing potential $\mathcal{U}_{\mathrm{eff}}$ which is not of importance for determining $\xi(T)$ from the OP.

In Figure 3a, we show the normal (G, $\bar{G}$) and anomalous (F) Green functions on imaginary time for different values of $q = |\boldsymbol{q}|$ where we also indicate the point of taking $|\Psi_q|$ at $\tau = 0^+$. The amplitude of F is reduced by increasing q, whereas G and $\bar{G}$ change only slightly. Interestingly, the anomalous Green function is a non-monotonous function of τ.

In the main text, we discuss the momentum-dependence of the OP obtained in DMFT calculations under the constraint of FMP. Here, we want to further elaborate on how $\xi(T)$ is

obtained from $\Psi_{\boldsymbol{q}}(T)$. In GL theory, we found that $\xi(T) = q_c^{-1}$ for $\lim_{q\to q_c} \Psi_q(T) = 0$. Since the point where Ψ_q goes to zero is difficult to evaluate numerically, we use in our DMFT calculations the criterion $|\Psi_{\boldsymbol{Q}}(T)/\Psi_0(T)| = 1/\sqrt{2}$ deriving from Eq. (5) such that $\xi(T) = 1/(\sqrt{2}|\boldsymbol{Q}|)$ for fixed T. Below, we test the robustness of this criterion by comparing it to other methods of numerically extracting $\xi(T)$ from $\Psi_{\boldsymbol{q}}$. In Figure 3b, we illustrate how this criterion is applied to the DMFT results. Since our microscopic calculations include higher order terms of the free energy, the exact momentum dependence of Ψ_q differs from the GL expectation (Eq. (5)). Note that we take $\xi(T)$ to be isotropic due to the high symmetry of the fcc lattice. In principle, it is possible to apply FMP with $\boldsymbol{q}$ in different directions in order to consider anisotropic behavior of $\xi(T)$.

Fig. 3 in the main text and Figure 3 here in the Supplementary Information show error bars for $\xi(T)$ which result from propagating the statistical QMC error of the OP to $\xi(T)$. The uncertainty in $\xi(T)$ has been estimated as follows: For every dataset $|\Psi_q(T)|$ we perform a series of spline fits where we randomly vary for each q the values to be fit in the range of $[|\Psi_q| - \delta|\Psi_q|, |\Psi_q| + \delta|\Psi_q|]$ spanned by the uncertainty $\delta|\Psi_q|$ of the OP. We indicate this range by color-shaded areas in Figure 3b. Based on each spline interpolation, we obtain a value for Q. The error in Q is then estimated as the standard deviation of Q values in the so-obtained ensemble.

The temperature dependence of extracted $\xi(T)$ and their uncertainty is plotted in panel c of Figure 3. As expected from GL theory, the correlation length diverges towards the critical temperature T_c and decays to a finite value ξ_0 for $T \to 0$. By fitting Eq. (3) to the data, we can extract the coherence length ξ_0 and also obtain a value for the critical temperature T_c. We discuss the utility of extracting T_c this way in Note 5-C.

Comparison of extraction methods for $\xi(T)$

As we conclude this section, we want to address the robustness of our criterion for extracting $\xi(T)$ from the q-dependence of the order parameter. To this end, we compare different methods of evaluating $\xi(T)$ given by:

(i) **Suppression of order parameter**: Identify the momentum Q where $|\Psi_Q/\Psi_0| = 1/\sqrt{2}$ and use $\xi = 1/(\sqrt{2}Q)$.

(ii) **Curvature method**: Calculate ξ from the curvature of $f_q = |\Psi_q/\Psi_0|^2 \to 1 - \xi^2 q^2$ for $q \to 0$ using $\xi^2 = -(1/2)\partial_q^2 f_q|_{q=0}$.

(iii) **Ginzburg–Landau fitting**: Fit the square-root Ginzburg–Landau (GL) expression $|\Psi_q/\Psi_0| = \sqrt{1-\xi^2 q^2}$ (to data points close to q) to determine ξ.

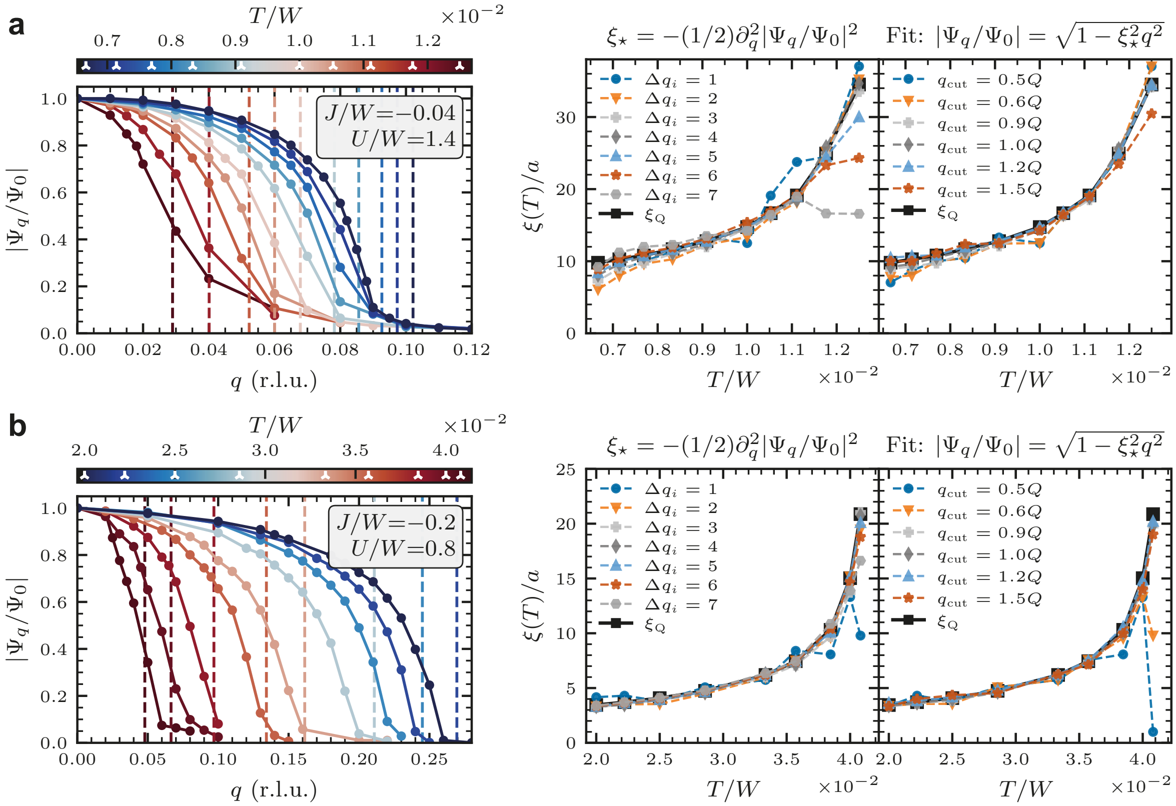

Supplementary Figure 4 | Comparison of methods for extracting the coherence length ξ. Panels (**a**) and (**b**) illustrate results for different values of J/W and U/W. The left column shows the normalized order parameter $|\Psi_q/\Psi_0|$ as a function of momentum q for various temperatures T, indicated by white triangles in the color bar. Dashed lines mark the momentum $\sqrt{2}Q = \xi_Q^{-1}$ which is associated with the coherence length ξ determined by method (i), see the text for labeling of methods. The right column contrasts ξ_Q (black squares) with different calculation methods for $\xi_\star$: (ii, left parts) evaluating the curvature of $f_q = |\Psi_q/\Psi_0|^2$ for different step sizes Δq_i, and (iii, right parts) fitting the Ginzburg–Landau expression (5) for different momentum cutoffs q_{cut}. Note that the step size Δq_i varies across temperatures due to differing q-meshes, which is why only the index i is listed in the legend (refer to the plots in the left column).

We compare the results for $\xi(T)$ using two sets of interaction parameters in Figure 4. The left column shows the momentum dependence of the normalized order parameter for various temperatures T (similar to Figure 3b). In addition, we marked the momentum $\xi_Q^{-1} = \sqrt{2}Q$ associated with the coherence length extracted from method (i). The right column compares ξ_Q (black squares) to $\xi_\star$ (colored points) obtained from methods (ii) and (iii) for different interval choices of numerical evaluation. For method (ii), we calculate the derivative using finite difference $\partial_q^2 f_q|_{q=0} \approx 2(f_{\Delta q} - f_0)/(\Delta q)^2$ and vary the step sizes $\Delta q_i = q_i - 0$ between 0.005 and 0.15 depending on T. Due to differing q-meshes for each temperature, we only list the

index i of the corresponding q-point (compare to data points in the $|\Psi_q/\Psi_0|$ plot). In approach (iii), the number of points included in the fit is controlled by q_{cut}. We tested different cutoffs relative to the Q-point from method (i).

Overall, we observe strong quantitative agreement across all methods. The comparison of the two parameter sets in panels a and b reveals no systematic trend of deviations from ξ_Q. We find the fitting (iii) to show less variance than calculating the curvature (ii). Intricacies with numerically evaluating second derivatives from numerical data are quite common. Among the tested methods, analyzing the suppression of the order parameter at momentum Q (i) has the advantage that it does not rely on fixing fitting intervals or step ranges for numerical derivatives, albeit it can pick up higher order effects in q. Therefore, we employ method (i) in this work.

5-B Current density and penetration depth

We derived in Note 3 an expression for the current density $\boldsymbol{j_q}$ (Eq. (26)) where we in practice employ the modified Eq. (40) to ensure better convergence of the Matsubara summations. We show results of $j_q = |\boldsymbol{j}_q|$ depending on the interaction value U/W for the *ab initio* estimated Hund's coupling value $J/W = -0.04$ and fixed $T/W = 6.7 \times 10^{-2}$ in Figure 5a. j_q exhibits a maximum, the depairing current j_{dp}, that we obtain by using a spline interpolation of the calculated data. By increasing U/W, j_{dp} exhibits a dome shape which is similar to the OP but different to T_{c}. We note that the momenta $q_{\max}$ where $j_{\mathrm{dp}} = j_{q_{\max}}$ correlate with the momenta Q used to calculate $\xi(T)$ from the OP suppression as can be seen in panel b. A line of slope $\sqrt{\frac{2}{3}}$ fits the data well suggesting $q_{\max} = \sqrt{\frac{2}{3}}Q$ as expected from the GL description. Only for large U, i.e., large values of Q and $q_{\max}$, deviations can be seen which arise from the fact that our DMFT calculations include higher order terms which are not accounted for in the GL expansion in Eq. (4).

From combining the depairing current and the coherence length, we obtain the London penetration depth $\lambda_{\mathrm{L}}(T)$. In GL theory, the T-dependence of λ_{L} is linearized to depend on $t = T/T_{\mathrm{c}}$. However, our calculations are better described by using the empirical quartic power law t^4 as stated in Eq. (11) (Eq. (3) of the main text). We show exemplary results of $\lambda_{\mathrm{L}}(T)$ for different U/W and $J/W = -0.04$ in Figure 5c. At small U, the t and t^4 dependence both match the data points quite well but the t-fit yields smaller values for the zero-temperature limit $\lambda_{\mathrm{L},0}$. Close to the Mott state for large U, the agreement becomes worse and only the t^4 dependence fits the data well. We observed the same behavior also in the strong coupling region for increased values of $|J|$.

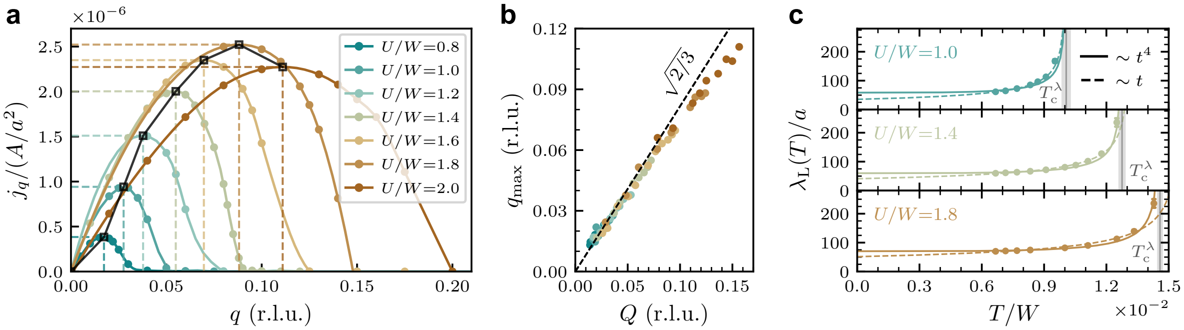

Supplementary Figure 5 | Evaluation of supercurrent density $j_q = |\boldsymbol{j}_q|$ and London penetration depth λ_L. (**a**) Momentum-dependence of the current density for different interaction ratios U/W with $J/W = -0.04$ similar to Fig. 3 of the main text and fixed $T/W = 6.7 \times 10^{-2}$. The depairing current density j_{dp} (maximal j_q) and corresponding momentum q_{max} are marked by dotted lines which are extracted from a spline interpolation drawn with a solid line connecting data points. The dome-shape behavior of j_{dp} as a function of U/W is marked by a black solid line. (**b**) Correlation between the momentum Q used to calculate $\xi(T)$ from the suppression of the OP (c.f. Note 5-A) and the momentum q_{max} of maximal current density j_{dp}. Data points of the same color correspond to different temperatures for the same U/W where the coloring is the same as in panel **a**. A linear function with slope $\sqrt{\frac{2}{3}}$ indicated by a dashed black line fits the data well. (**c**) Temperature dependence of the London penetration depth λ_L for different U/W and $J/W = -0.04$. We plot the fit according to Eq. (11) (Eq. (3) in the main text) with the quartic temperature dependence with a solid line and the fit with a linear temperature dependence with a dashed line ($t = T/T_c$).

5-C Proximity region to the Mott transition

From our analysis of the T-dependence of the zero-momentum OP $|\Psi_0|$, correlation length $\xi(T)$, and London penetration depth λ_L, we are able to obtain different values of the superconducting transition temperature T_c. In this section, we discuss how they compare and use the notation of $T_c^{\xi,\lambda}$ to differentiate the critical temperatures obtained by fitting $\xi(T)$ and $\lambda_L(T)$ from the T_c derived via $|\Psi_0|^2 \sim T - T_c$.

A first understanding can be gained by analyzing Fig. 3 of the main text. We summarize the respective critical temperatures in Figure 6a. Generally, the critical temperature values obtained in all three methods agree well. Only in the special case of the first-order transition from the superconducting to the Mott-insulating phase for $U/W = 2$, we obtain higher values for $T_c^{\xi,\lambda}$. We conjecture that these temperatures describe a second-order transition to a metallic state hidden by the Mott insulating phase. We can utilize this fact to gauge the influence of the Mott state to reveal a suppression of superconductivity.

In Figure 6b, we show the critical temperature T_c and the relative difference to $T_c^{\xi,\lambda}$ in the

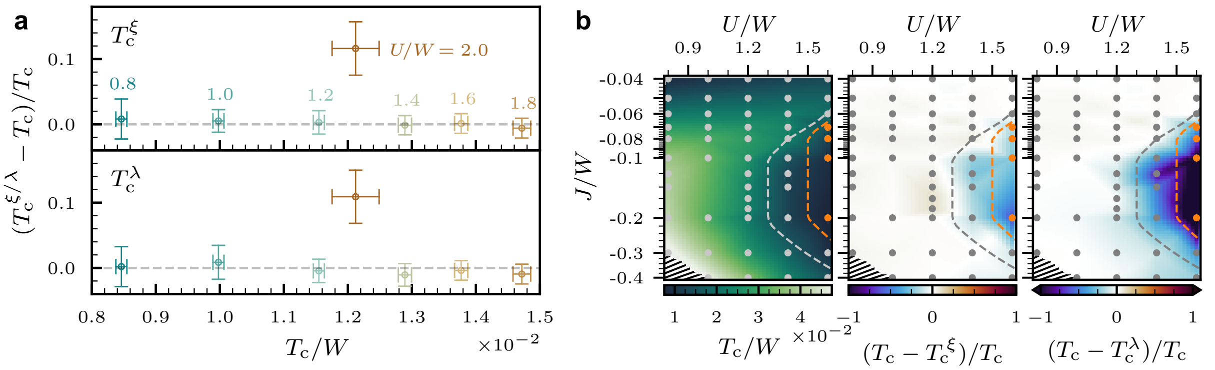

Supplementary Figure 6 | Influence of proximity to the Mott insulating region on the superconducting state. (**a**) Relative difference of critical temperature $T_c^{(\xi,\lambda)}$ computed from fitting $\xi(T)$ and $\lambda_L(T)$ versus T_c as obtained from the OP $|\Psi_0|$ for the data at $J/W = -0.04$ and different U/W as plotted in Fig. 3 of the main text. (**b**) T_c and relative difference to $T_c^{(\xi,\lambda)}$ as a function of interactions U and J. Orange (gray) dots indicate data points where the critical temperature describes a transition from superconducting to Mott insulating (metallic) phase. Dashed lines are a guide to the eye separating the regions where the proximity to the Mott phase suppresses superconductivity characterized by $T_c^{(\xi,\lambda)} < T_c$. The T_c plot is the same as in Fig. 4a of the main text.

(U, J)-plane analogous to Fig. 4a of the main text. Dots indicate original data points where orange dots (not shown in Fig. 4a) denote a critical temperature for a first-order transition from superconductor to Mott insulator. At these points, both T_c^ξ and T_c^λ are clearly larger than the critical temperature obtained from $|\Psi_0|^2$ which is inline with the observation at $J/W = -0.04$. However, the suppression of T_c extends to the nearby region of the direct superconductor–Mott transition. The dashed lines, of which we also draw the gray line in Fig. 4a of the main text, are a guide to the eye to separate the region where proximity to Mott insulating states leads to a suppression of the critical temperature – even for a transition to the metallic state.

Supplementary Note 6: Analysis of superconducting gap and coupling strength

In this section, we analyze the superconducting gap Δ to further characterize the different superconducting regimes found in the A_3C_{60} model. The gap is given by [103]

$$\Delta(i\omega_n) = \frac{\mathrm{Re}\Sigma^{\mathrm{AN}}(i\omega_n)}{\mathbb{1} - \frac{\mathrm{Im}\Sigma^{\mathrm{N}}(i\omega_n)}{\omega_n}} \equiv Z\Sigma^{\mathrm{AN}} \tag{51}$$

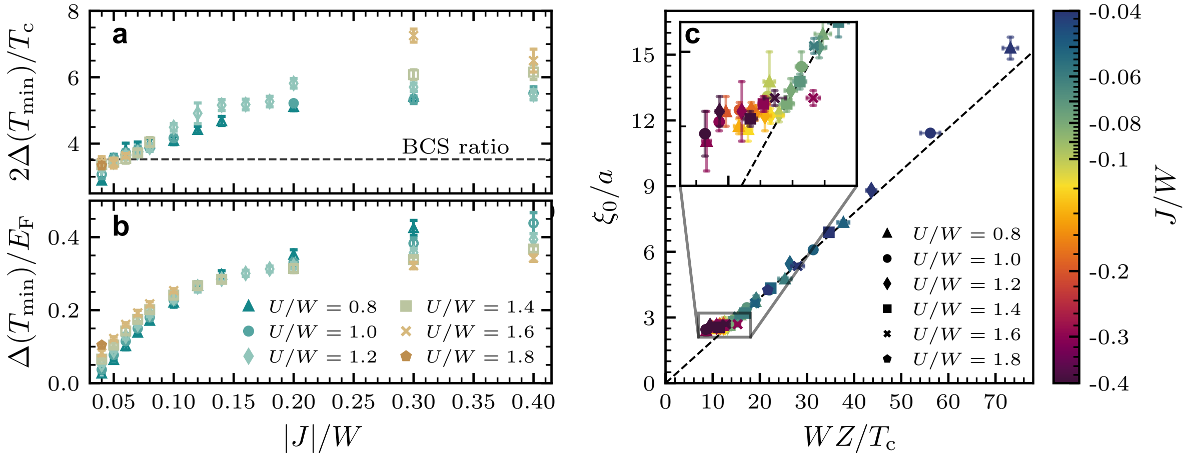

Supplementary Figure 7 | Characterization of superconducting regions in the model of A_3C_{60}. (**a**) Ratio of the superconducting gap Δ at the lowest available temperature $T_{\min}$ and critical temperature T_c as a function of inverted Hund's coupling $J < 0$. The BCS ratio $\Delta_0/T_c = 3.53$ is drawn with a dashed line. (**b**) Coupling strength characterized by the ratio of Δ against the Fermi energy E_F. (**c**) Scaling of the coherence length ξ_0 with the ratio of quasiparticle weight Z and T_c. Note that the color scale used to mark Hund's coupling $J < 0$ is logarithmic. A linear fit is drawn as a guide to the eye where the zoom-in shows deviations for large $J < 0$. We note that T_c shown in panels **a** and **c** are only for the superconductor-metal transition, not for a first-order transition to the Mott phase.

with the quasiparticle weight $Z^{-1} = \mathbb{1} - \mathrm{Im}\Sigma^{\mathrm{N}}(i\omega_0)/\omega_0$ and anomalous self-energy Σ^{AN} as we evaluate the gap on the lowest Matsubara frequency $\Delta \equiv \Delta(i\omega_0)$. We note that in the (deep) BEC limit, the quasiparticle gap is not given by Δ due to the appearance of bound bosonic states. Instead, one needs to analyze $\sqrt{\Delta^2 + \mu^2}$ with the chemical potential μ.

In order to characterize the superconducting state, we analyze two different criteria: The first is the BCS ratio of the gap to the critical temperature T_c which in weak-coupling BCS theory has the universal value $2\Delta_0/T_c = 3.53$ for the zero-temperature gap $\Delta_0 = \Delta(T = 0)$. In our calculations, we cannot reach zero temperature because of which we consider the gap for the lowest temperature $T_{\min}$ available for each interaction parameter set (U, J). This is important for interpreting results for small Hund's coupling $J/W \lesssim -0.1$ where we could not calculate far below T_c, i.e, the gap is far from saturating towards the zero-temperature value. Hence, $\Delta(T_{\min})$ only yields a lower bound.

We show the BCS ratio in Figure 7a as a function of inverted Hund's coupling strength $J < 0$ for different U/W. For small magnitudes $|J|/W \lesssim 0.05$, our results show good agreement with the BCS value. This is in disagreement with experimental measurements [36, 80] which observe the Cs and Rb compounds to have a ratio $2\Delta/T_c$ much larger than the BCS value. We

speculate that the discrepancy to our data arises for two reasons: First, we cannot get close to the zero-temperature value of the gap in our calculations, i.e., T_{min} is rather close to T_{c} and the ratio is likely to be underestimated by this lower bound. Second, the overestimation of T_{c} in DMFT can additionally lead to an underestimation of the BCS ratio. Taking into account dynamical interactions give results that are in better agreement with experiment [67, 68]. Nonetheless, the qualitative trend of increasing $2\Delta/T_{\text{c}}$ for larger U/W fits to experimental observation. A pronounced deviation from the BCS value can be found for large inverted Hund's coupling $|J|/W > 0.05$. Although T_{c} and Δ both increase in the "multiorbital strong coupling" region for enhanced $J < 0$, superconductivity here is distinct to weak-coupling BCS theory. Note that we do not show T_{c} of a transition to the Mott insulating state.

The second criterion that we analyze is the ratio of the gap to the Fermi energy E_{F}. This ratio Δ/E_{F} can be interpreted as a dimensionless coupling strength [25] which is small in the weak-coupling region but grows to the order of $0.1 - 1$ in the crossover and strong-coupling regime [24]. We note, however, that the theoretical determination of E_{F} is not trivial. To gauge the order of magnitude, we here resort to the non-interacting, renormalized definition

$$E_{\text{F}} = k_{\text{B}}T_{\text{F}} = \frac{\hbar^2 k_{\text{F}}^2}{2m^*} = \frac{\hbar^2}{2m^*}(3\pi^2 n)^{\frac{2}{3}} = \frac{(3\pi^2)^{\frac{2}{3}}\hbar^2}{2m_e} \cdot Zn^{\frac{2}{3}} \tag{52}$$

where we inserted the quasiparticle weight $Z = m^*/m_e$. The density is $n = 3/(a/4)^3$ for the half-filled $t_{1\text{u}}$ bands. We show the results in Figure 7b. For small $|J|$, the coupling strength is weak as $\Delta/E_{\text{F}} < 0.1$. Towards the Mott regime, the couplings strengths grows to ~ 0.1, i.e., increasing U/W brings the system towards the BCS–BEC crossover regime. However, increasing $|J|$ has a much stronger effect of driving the system into a strong coupling phase with $\Delta/E_{\text{F}} > 0.1$. Interestingly, larger U here quenches the coupling strength.

Lastly, we want to characterize the superconducting regimes via the coherence length ξ_0. In BCS theory and Eliashberg theory, the scaling $\xi_0 \sim v_{\text{F}}^*/T_{\text{c}} \propto Z/T_{\text{c}}$ in terms of a renormalized Fermi velocity $v_{\text{F}}^* = Zv_{\text{F}}$ can be established [39–41]. We investigate this relation in Figure 7c. The scaling $\xi_0 \sim Z/T_{\text{c}}$ as in BCS and Eliashberg theory holds for most interaction values up to $J/W \sim -0.1$, even towards the Mott insulating region. It, however, deviates in the localized multiorbital strong coupling region for large $|J|/W > 0.1$, where ξ_0 is not further reduced and Z stays almost constant but T_{c} still increases. Thus, the multiorbital strong coupling phase is to be differentiated from a (strongly) renormalized Eliashberg system as which one might be able to describe the system in the vicinity of the Mott insulating phase.

Supplementary Note 7: Atomic limit of three-orbital model with inverted Hund's coupling

In the main text, we found that Cooper pairs become very localized with a short coherence length $\xi_0 \sim \mathcal{O}(2-3\,a)$ by increasing the inverted Hund's coupling $J < 0$. It suggests that local physics become increasingly important for the formation of superconducting pairing. Indeed, this was confirmed through the analysis of local density matrix weights in the main text. Here, we want to complement the discussion of the main text with discussing the atomic limit of the interacting impurity problem.

To this end, we want to solve the Kanamori–Hubbard interaction Hamiltonian as given in Eq. (7) of the main text without hopping processes. The form of the interaction given in the main text is convenient to read-off the (inverted) Hund's rules. We, here, restate the Kanamori–Hubbard Hamiltonian in its generalized formulation that indicates the different electronic interaction processes more clearly:

$$
\begin{aligned}
H_{\mathrm{int}} = &\sum_{\alpha} U\, n_{\alpha\uparrow} n_{\alpha\downarrow} + \sum_{\alpha<\gamma,\sigma\sigma'} (U' - \delta_{\sigma\sigma'} J)\, n_{\alpha\sigma} n_{\gamma\sigma'} \\
&- \sum_{\alpha\neq\gamma} J_{\mathrm{X}}\, c^{\dagger}_{\alpha\uparrow} c_{\alpha\downarrow} c^{\dagger}_{\gamma\downarrow} c_{\gamma\uparrow} + \sum_{\alpha\neq\gamma} J_{\mathrm{P}}\, c^{\dagger}_{\alpha\uparrow} c^{\dagger}_{\alpha\downarrow} c_{\gamma\downarrow} c_{\gamma\uparrow}
\end{aligned} \tag{53}
$$

It consists of intraorbital interaction U, interorbital interaction U', Hund's coupling J, spin-exchange J_{X}, and correlated pair hopping J_{P}. Yet, not all coupling constants are independent. We assume SU(2)×SO(3) symmetry implying $J_{\mathrm{X}} = J$ and $J_{\mathrm{P}} = U - U' - J$ [69]. In the physical system and our calculations, we have in addition $J_{\mathrm{P}} = J$ resulting in $U' = U - 2J$. In the following discussion, we will emphasize the contribution of J_{P} since the low-energy excitations for inverted Hund's coupling $J < 0$ are only governed by J_{P}. It is instructive to rewrite Eq. (53) in the same way as Eq. (7) of the main text (c.f. Eq. 5 in Ref. [69]) to see the role of J_{P}:

$$
H_{\mathrm{int}} = \frac{1}{4}(2U - 3J - 3J_{\mathrm{P}})\hat{N}(\hat{N}-1) - (J + J_{\mathrm{P}})\hat{\boldsymbol{S}}^2 - \frac{1}{2}J_{\mathrm{P}}\hat{\boldsymbol{L}}^2 + \frac{1}{4}(3J + 7J_{\mathrm{P}})\hat{N} \tag{54}
$$

The pair hopping term, most notably, dictates the energy gain from high orbital angular momentum L^2 and partially that of the total spin S^2 of a given eigenstate for this Hamiltonian. We detail the spectrum in Tab. 1 for the case of negative $J, J_{\mathrm{P}} < 0$ and half-filled orbitals where we add a chemical potential μ to ensure particle-hole symmetry. The chemical potential at half-filling is given by $\mu = \frac{1}{2}U + \frac{N-1}{2}(2U' - J) = \frac{5}{2}U - 3J - 2J_{\mathrm{P}}$ with $N = 3$ and $U' = U - J - J_{\mathrm{P}}$, as can be inferred from particle-hole transforming Eq. (54). The dimension of the complete Fock space is $\dim \mathcal{H}_{\mathrm{Fock}} = 2^6 = 64$. Fig. 4b in the main text shows the statistical occupation of these 64 states during the QMC calculation in DMFT.

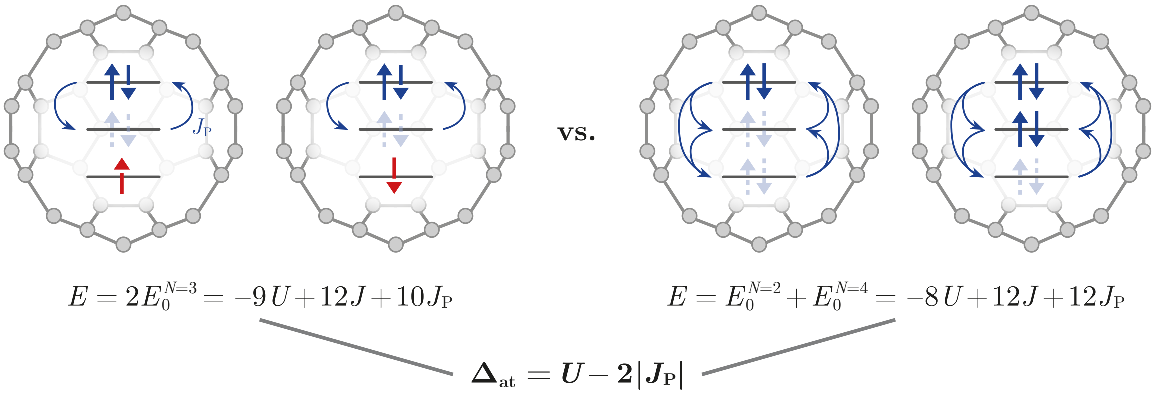

Supplementary Figure 8 | Schematic energy diagram of the atomic gap Δ_{at}. The left and right sides show the charge configurations (3,3) and (2,4) of neighboring C_{60} molecules, respectively, whose energy difference is given by Δ_{at}.

In case of half-filling, the atomic gap of the system is $\Delta_{at} = E_0^{N=4} - E_0^{N=3} - (E_0^{N=3} - E_0^{N=2}) = U + 2J_P = U - 2|J_P|$, see Figure 8. For $\Delta_{at} > 0$, i.e., $U > 2|J_P|$, the lowest energy state lies in the $N = 3$ particle sector and is given by $E_0^{N=3}$. We sketch the energy spectrum for this case in Figure 9. The lowest energy excitations from the ground state $E_0^{N=3}$ are charge excitation to the $N = 2$ and $N = 4$ particle sectors with $\Delta E_{ch} = \frac{1}{2}U + J_P = \frac{1}{2}U - |J_P| \equiv \frac{1}{2}\Delta_{at}$ as well as spin reconfiguration with $\Delta E_{sp} = -2J_P = 2|J_P|$ within the $N = 3$ charge sector which breaks up orbital singlets and increases the orbital angular momentum from $L = 1$ to $L = 2$. Thus, the low-energy physics is governed by correlated pair hopping J_P and onsite repulsion U. The results presented in Fig. 4 of the main text can be understood from this local limit by addition of the kinetic hopping. In this work, we do not address the regime of charge disproportionation for $\Delta_{at} < 0$ (i.e. $U < 2|J_P|$), where the $N = 3$ particle sector is at a higher energy level compared to the $N = 2$ and $N = 4$ sectors [82].

References

101. Kim, D.-H., Kinnunen, J. J., Martikainen, J.-P. & Törmä, P. Exotic Superfluid States of Lattice Fermions in Elongated Traps. *Phys. Rev. Lett.* **106,** 095301. doi:10.1103/physrevlett.106.095301 (2011).

102. Heikkinen, M., Kim, D.-H., Troyer, M. & Törmä, P. Nonlocal Quantum Fluctuations and Fermionic Superfluidity in the Imbalanced Attractive Hubbard Model. *Phys. Rev. Lett.* **113,** 185301. doi:10.1103/physrevlett.113.185301 (2014).

103. Gull, E. & Millis, A. J. Pairing glue in the two-dimensional Hubbard model. *Phys. Rev. B* **90,** 041110. doi:10.1103/physrevb.90.041110 (2014).

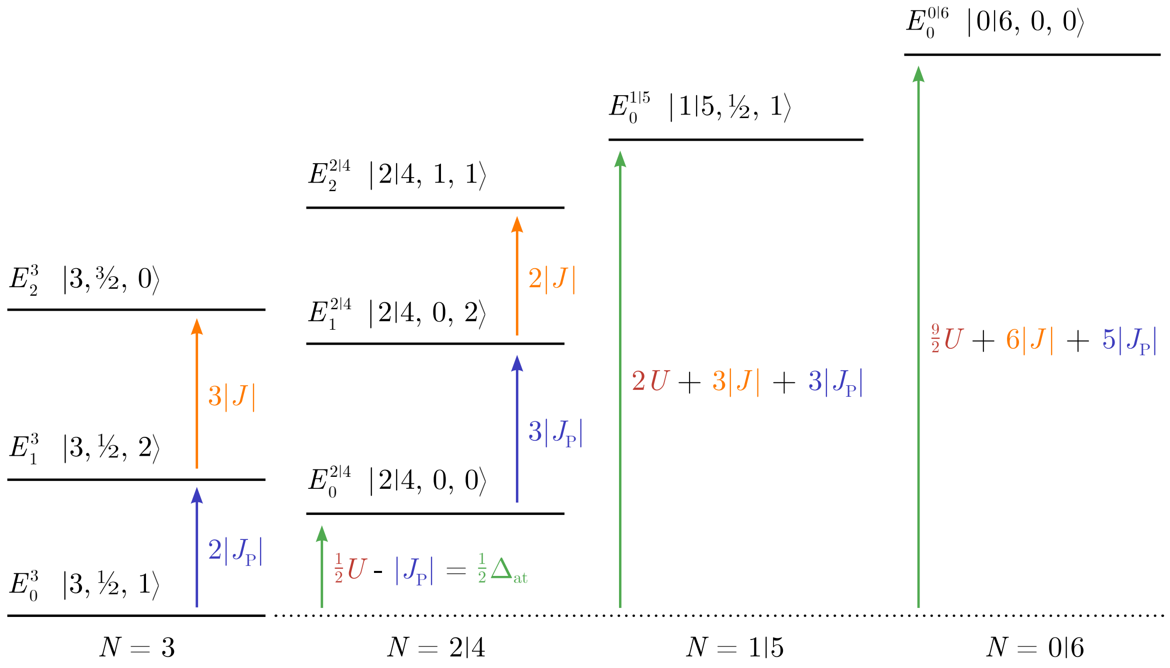

Supplementary Figure 9 | Spectrum of the three-orbital Kanamori–Hubbard model. The energies E_n^N and states $|\phi_n^N\rangle \equiv |N, S, L\rangle$ of the three-orbital atom with Kanamori–Hubbard interaction (Eq. (54) at half-filling correspond to the notation used in Tab. 1. The relative positions of energies are drawn for the case of $U > 2|J_P|$, such that the ground state lies in the $N = 3$ sector given by $E_0^{N=3}$ and $|N = 3, S = \frac{1}{2}, L = 1\rangle$.

Supplementary Table 1 | Spectrum of the local Kanamori–Hubbard Hamiltonian for a three-orbital system at half-filling. The eigenenergies E_n^N are sorted in descending order in each charge sector of particle number N for $J < 0$ where the contribution of the correlated pair hopping J_P ($J_\mathrm{P} \equiv J$ in our calculations) is explicitly stated. Each state is characterized by total spin S, orbital angular momentum L, and respective degeneracy $(2S+1)(2L+1)$ with $X = \langle \hat{X} \rangle$ $(X = N, S, L)$. The corresponding eigenstates $|\phi_n^N\rangle$ are given for $N \leq 3$ since the $N \geq 4$ states can be constructed from particle-hole symmetry. The eigenstates in blue color are those depicted in Fig. 4c of the main text.

Energy E_n^N	N	S	L	Degeneracy $(2S+1)(2L+1)$	Eigenstates $\vert\phi_n^N\rangle$ $(N \leq 3)$
$E_0^{0\,[6]} = 0$	0 [6]	0	0	1	$\vert 0,0,0\rangle$
$E_0^{1\,[5]} = -\frac{5}{2}U + 3J + 2J_\mathrm{P}$	1 [5]	1/2	1	6	$\vert\uparrow,0,0\rangle$, $\vert 0,\uparrow,0\rangle$, $\vert 0,0,\uparrow\rangle$,<br>$\vert\downarrow,0,0\rangle$, $\vert 0,\downarrow,0\rangle$, $\vert 0,0,\downarrow\rangle$
$E_2^{2\,[4]} = -4U + 4J + 3J_\mathrm{P}$	2 [4]	1	1	9	$\vert\uparrow,\uparrow,0\rangle$, $\vert 0,\uparrow,\uparrow\rangle$, $\vert\uparrow,0,\uparrow\rangle$, $\vert\downarrow,0,\downarrow\rangle$, $\vert 0,\downarrow,\downarrow\rangle$, $\vert\downarrow,0,\downarrow\rangle$,<br>$\frac{1}{\sqrt{2}}(\vert\uparrow,0,\downarrow\rangle - \vert\downarrow,0,\uparrow\rangle)$, $\frac{1}{\sqrt{2}}(\vert 0,\uparrow,\downarrow\rangle - \vert 0,\downarrow,\uparrow\rangle)$, $\frac{1}{\sqrt{2}}(\vert\uparrow,\downarrow,0\rangle - \vert\downarrow,\uparrow,0\rangle)$
$E_1^{2\,[4]} = -4U + 6J + 3J_\mathrm{P}$	2 [4]	0	2	5	$\frac{1}{\sqrt{2}}(\vert\uparrow,0,\downarrow\rangle + \vert\downarrow,0,\uparrow\rangle)$, $\frac{1}{\sqrt{2}}(\vert 0,\uparrow,\downarrow\rangle + \vert 0,\downarrow,\uparrow\rangle)$, $\frac{1}{\sqrt{2}}(\vert\uparrow,\downarrow,0\rangle + \vert\downarrow,\uparrow,0\rangle)$<br>$\frac{1}{\sqrt{2}}(\vert\uparrow\downarrow,0,0\rangle - \vert 0,\uparrow\downarrow,0\rangle)$, $\frac{1}{\sqrt{2}}(\vert\uparrow\downarrow,0,0\rangle - \vert 0,0,\uparrow\downarrow\rangle)$
$E_0^{2\,[4]} = -4U + 6J + 6J_\mathrm{P}$	2 [4]	0	0	1	$\frac{1}{\sqrt{3}}(\vert\uparrow\downarrow,0,0\rangle + \vert 0,\uparrow\downarrow,0\rangle + \vert 0,0,\uparrow\downarrow\rangle)$
$E_2^3 = -\frac{9}{2}U + 3J + 3J_\mathrm{P}$	3	3/2	0	4	$\vert\uparrow,\uparrow,\uparrow\rangle$, $\frac{1}{\sqrt{3}}(\vert\uparrow,\downarrow,\downarrow\rangle + \vert\downarrow,\uparrow,\downarrow\rangle + \vert\downarrow,\downarrow,\uparrow\rangle)$,<br>$\vert\downarrow,\downarrow,\downarrow\rangle$, $\frac{1}{\sqrt{3}}(\vert\downarrow,\uparrow,\uparrow\rangle + \vert\uparrow,\downarrow,\uparrow\rangle + \vert\uparrow,\uparrow,\downarrow\rangle)$
$E_1^3 = -\frac{9}{2}U + 6J + 3J_\mathrm{P}$	3	1/2	2	10	$\frac{1}{\sqrt{2}}(\vert\uparrow,\downarrow,\downarrow\rangle - \vert\downarrow,\uparrow,\downarrow\rangle)$, $\frac{1}{\sqrt{2}}(\vert\uparrow,\downarrow,\downarrow\rangle - \vert\downarrow,\downarrow,\uparrow\rangle)$,<br>$\frac{1}{\sqrt{2}}(\vert\uparrow,\uparrow\downarrow,0\rangle - \vert\uparrow,0,\uparrow\downarrow\rangle)$, $\frac{1}{\sqrt{2}}(\vert\uparrow\downarrow,\uparrow,0\rangle - \vert 0,\uparrow,\uparrow\downarrow\rangle)$, $\frac{1}{\sqrt{2}}(\vert\uparrow\downarrow,0,\uparrow\rangle - \vert 0,\uparrow\downarrow,\uparrow\rangle)$,<br>$\frac{1}{\sqrt{2}}(\vert\downarrow,\uparrow\downarrow,0\rangle - \vert\downarrow,0,\uparrow\downarrow\rangle)$, $\frac{1}{\sqrt{2}}(\vert\uparrow\downarrow,\downarrow,0\rangle - \vert 0,\downarrow,\uparrow\downarrow\rangle)$, $\frac{1}{\sqrt{2}}(\vert\uparrow\downarrow,0,\downarrow\rangle - \vert 0,\uparrow\downarrow,\downarrow\rangle)$,<br>$\frac{1}{\sqrt{2}}(\vert\downarrow,\uparrow,\uparrow\rangle - \vert\uparrow,\downarrow,\uparrow\rangle)$, $\frac{1}{\sqrt{2}}(\vert\downarrow,\uparrow,\uparrow\rangle - \vert\uparrow,\uparrow,\downarrow\rangle)$
$E_0^3 = -\frac{9}{2}U + 6J + 5J_\mathrm{P}$	3	1/2	1	6	$\frac{1}{\sqrt{2}}(\vert\uparrow,\uparrow\downarrow,0\rangle + \vert\uparrow,0,\uparrow\downarrow\rangle)$, $\frac{1}{\sqrt{2}}(\vert\uparrow\downarrow,\uparrow,0\rangle + \vert 0,\uparrow,\uparrow\downarrow\rangle)$, $\frac{1}{\sqrt{2}}(\vert\uparrow\downarrow,0,\uparrow\rangle + \vert 0,\uparrow\downarrow,\uparrow\rangle)$,<br>$\frac{1}{\sqrt{2}}(\vert\downarrow,\uparrow\downarrow,0\rangle + \vert\downarrow,0,\uparrow\downarrow\rangle)$, $\frac{1}{\sqrt{2}}(\vert\uparrow\downarrow,\downarrow,0\rangle + \vert 0,\downarrow,\uparrow\downarrow\rangle)$, $\frac{1}{\sqrt{2}}(\vert\uparrow\downarrow,0,\downarrow\rangle + \vert 0,\uparrow\downarrow,\downarrow\rangle)$
